# G-protein coupled receptors synergy in bone health: new avenues for osteoporosis detection and *in vitro* modeling

**DOI:** 10.3389/fendo.2025.1684658

**Published:** 2025-11-27

**Authors:** Julia V. Sopova, Olga A. Krasnova, Julia D. Kriukova, Yana O. Mukhamedshina, Elena Y. Zakirova, Albert A. Rizvanov, Olga M. Lesnyak, Irina E. Neganova

**Affiliations:** 1 Institute of Cytology, Russian Academy of Sciences, Saint-Petersburg, Russia; 2 Center for Transgenesis and Genome Editing, Saint-Petersburg State University, Saint-Petersburg, Russia; 3 Institute of Fundamental Medicine and Biology, Kazan (Volga Region) Federal University, Kazan, Russia; 4 Division of Medical and Biological Sciences, Academy of Sciences of the Republic of Tatarstan, Kazan, Russia; 5 Department of Family Medicine, North-Western State Medical University Named After I.I. Mechnikov, St. Petersburg, Russia

**Keywords:** osteoporosis, SNP, bone remodeling, bone homeostasis, FSHR, TSHR, ADRB2

## Abstract

Osteoporosis remains a substantial healthcare burden in modern times. Current diagnostic methods of osteoporosis detect changes in bone mineral density and microarchitecture, which have already occurred. It is critically important to develop methods of early diagnosis of osteoporosis to be able to plan early interventions in order to stop the disease progression. Genetic screening based on early osteoporosis marker genes appears to be a promising approach for early diagnosis and prevention. However, a significant gap exists in this area of knowledge. Recently, we identified a novel combination of three single nucleotide polymorphisms – *FSHR* (rs6166) AA, *TSHR* (rs1991517) CC, and *ADRB2* (rs1042713) AA, with a high prevalence among osteoporotic patients. Subsequent functional studies using patient-derived mesenchymal stem cell lines revealed impaired osteogenic differentiation capacity. To clarify the role of these polymorphism combinations, this review first examines the physiological aspects of each receptor and the identified single nucleotide polymorphisms at the organismal level. It then analyzes their contribution to the dysregulation of bone remodeling, with a particular focus on osteoblastogenesis. Understanding these mechanisms opens up new opportunities for the development of early osteoporosis diagnosis and stratification of personalized treatments for patients.

## 1 Introduction

Members of the G protein-coupled receptors (GPCRs) superfamily mediate diverse biological processes upon activation by extracellular signals. Their important role in bone development, remodeling, and osteopathologies is well established. To date, mutations in approximately 36 GPCRs have been recognized as causative for bone tissue dysfunction in humans, with several of these mutations being associated with low bone mineral density (BMD) – a major risk factor for osteoporosis (1). The connection between altered BMD and osteoporosis risk has been further supported by numerous population genetic studies. This includes genome-wide association studies (GWAS), which have identified correlations between osteoporotic phenotypes and various single nucleotide polymorphisms (SNPs) (2). Although low BMD is a strong predictor of osteoporotic fractures, it is considered a late-stage marker, as it reflects structural and density changes in bone that have already occurred, indicating disease progression.

Low BMD level and defective bone microarchitecture reflect the functional status of bone cells. Bone homeostasis as a dynamic system, regulated by a balance between action of bone resorptive cells – osteoclasts and new bone formation driven by osteoblast. This fine equilibrium between bone formation and resorption is disrupted in osteoporosis. In addition, osteocytes, terminally differentiated osteoblasts, serve as critical regulators of bone homeostasis by controlling osteoblast and osteoclast activity.

Advances in GWAS have identified hundreds of susceptibility loci for osteoporosis and low BMD, for example, mutations in genes *CLCN7*, *GALNT3*, *IBSP*, *LTBP3*, *RSPO3*, and *SOX4*. However, approximately 80% of these loci are located in non-coding genomic regions, providing only limited insight into disease mechanism and genetics. Nevertheless, low BMD and osteoporosis share many common susceptibility loci, which are significantly enriched in biological pathways such as Wnt/β-catenin, Notch, NF-κB, BMP and other pathways, known to be crucial for bone health (3). Activation of the canonical Wnt/β-catenin signalling pathway in osteoblasts and osteocytes increases bone mass by reducing bone resorption, thus making it one of the central for bone homeostasis and bone anabolism (4). The RANKL- RANK–OPG system is a complex signalling pathway that exerts crucial effects on the MSCs osteogenic differentiation and plays a critical role in bone metabolism. The receptor activator of the nuclear factor-κB ligand (RANKL) is a member of the tumor necrosis factor-α (TNF-α) superfamily. This ligand binds to RANK, a receptor expressed on osteoclasts. RANKL signalling promotes osteoclast differentiation and activation, thus leading to bone resorption. Osteoprotegerin (OPG) is also a member of the TNF receptor superfamily and acts as a decoy receptor to RANKL, inhibiting the activation of RANK signalling and thereby limiting osteoclast formation. Gene variants of the members of this signalling pathway, namely in RANKL, RANK, OPG, TRAF6, and NFATC1, are found not only to be associated with altered BMD level but also correlate with the increased incidence of osteoporotic fractures (5).

GWAS have identified 56 BMD-associated loci, approximately 15 of which are also linked to fracture risk. Among the most well-recognized are loci coding for Wnt pathway components associated with low BMD (*CTNNB1*, *SOST*, *LRP4*, *LRP5*, *WLS*, *WNT4*, and *MEF2C)*, as well as loci implicated in Wnt signalling, such as *WNT5B*, *WNT16*, *DKK1*, *PTHLH*, *SFRP4*, and *AXIN1*. These data highlight crucial role of Wnt/β-catenin and Hippo as regulators of skeletal development, homeostasis, and repair. The crosstalk of these two pathways is recognized as a fundamental mechanism of bone development. Dysregulation in these pathways provides some of the earliest genetic markers for bone disease. Loss-of-function mutations in LRP5 decrease signalling through Wnt pathway, leading to osteoporosis. Inactivating mutations in *LRP5* cause Osteoporosis-Pseudoglioma Syndrome (OPPG**),** presenting with early-onset osteoporosis and blindness. It is a clear genetic marker for severe, juvenile osteoporosis. Similarly, *LRP6* is a Wnt co-receptor. Its mutations are less common but are linked to coronary artery disease and metabolic syndrome, which can have skeletal complications, including early-onset osteoporosis (6). The *SOST* gene encodes sclerostin, a protein secreted by osteocytes, which inhibits Wnt/β-catenin signalling by binding to LRP5/6. Loss of sclerostin leads to unchecked bone formation (7). AXIN2 is a component of the β-catenin destruction complex. Mutations in *AXIN2* lead to the formation of stabilized β-catenin and overactive signalling. These mutations are linked to familial tooth agenesis and a predisposition to colorectal cancer. Axin2 deficiency is a specific marker for the oral bone (mandible/maxilla) specific genetic syndrome (8). Hippo pathway controls cell fate by regulating the transcriptional co-activators YAP (Yes-associated protein) and TAZ (Transcriptional co-activator with PDZ-binding motif, encoded by *WWTR1*). In bone, YAP/TAZ activity is critical for determining mesenchymal stem cell (MSC) fate, specifically, promoting osteogenesis over adipogenesis. The expression and nuclear localization of YAP/TAZ are among early markers of osteogenic differentiation. Both YAP and TAZ are often overexpressed and hyperactive in osteosarcoma (9, 10). The molecular mechanism of how YAP/TAZ integrate into the Wnt pathway is discussed in (11). In osteoporosis, reduced Wnt signalling may be compounded by inadequate YAP/TAZ activity in response to mechanical unloading (11). As highlighted above, genes of the Wnt/β-catenin pathway, like *LRP5*, *LRP4, SOST*, serve as some of the most well-defined genetic markers for inherited bone density disorders. In contrast, the Hippo pathway effectors YAP/TAZ are more relevant as somatic markers of bone cell fate and cancer progression. In the nucleus, YAP/TAZ complexed with TEADs and β-catenin, complexed with TCF/LEFs, forms multimolecular complexes with other factors to co-occupy the enhancers/promoters of key osteogenic genes, such as for example, *RUNX2*, leading to synergistic activation. The interplay between these two Hippo and Wnt pathways is now considered to be a trigger for genetic and molecular events in both congenital and acquired bone diseases (12, 13). Genes involved in MSCs osteogenic differentiation include *RUNX2*, *SOX4*, *SOX9*, and *SP7*. Most of genes in this list are also implicated in promoting endochondral ossification, an essential process during fetal skeletal development (this includes *SPP1*, *MEF2C*, *RUNX2*, *SOX6*, *PTHLH*, *SP7*, and *SOX9)*. Additionally, genes associated with rare monogenic forms of osteoporosis, such as *SOST*, *CLCN7*, and *LRP5*, have also been reported to influence BMD variation in diverse population studies (2, 14, 15).

Osteoporosis depends upon the cross-talks between several signalling pathways. Impairment of this pathway cross-talk leads to pathology. Canonically, Wnt/b-catenin, Notch, and NF-κB are considered to bethe main players in this cross-talk. However, signalling via PTHrP, TGF-β, BMP, FGF, Sonic Hedgehog and others less-studied pathways may be involved. The functional role of multiple converging pathways for bone homeostasis has been widely discussed (16–19). Besides that, several papers and reviews discuss the bone-specific role of signalling pathways associated with inflammation, oxidative stress, cellular senescence, epigenetic factors and factors associated with various comorbid diseases (20–23).

Practically, low BMD remains one of the key predictors of fracture risk. However, in light of the fact that osteoporosis becomes a pandemic, there is a pressing need to identify early genetic markers of the disease.

Recently, our study of postmenopausal women diagnosed with osteoporosis revealed a novel combination of three SNPs in GPCR genes — *FSHR* (rs6166), *TSHR* (rs1991517), and *ADRB2* (rs1042713) — with a high frequency of approximately 20% (24). Follow-up studies using patient-specific cell lines confirmed a reduced osteogenic differentiation potential and impaired bone matrix mineralization of MSCs derived from bone biopsies (24, 25). Although missense mutations in *FSHR*, *TSHR*, and *ADRB2* have previously been associated with reduced BMD and osteoporosis (26–28), the combined effect of these specific SNPs has not yet been considered as a potential prognostic marker.

In this review, we aim to discuss the interaction and regulation of the identified receptors at the level of the human body (*in vivo*), link this knowledge with the *in vitro* cellular models of osteoporosis alongside with data generated via animal studies and explore the potential of this new SNPs combination as a novel marker for osteoporosis diagnostic.

## 2 FSH- FSHR biology

Follicle-stimulating hormone (FSH) belongs to a family of glycoprotein hormones that includes luteinizing hormone (LH), thyroid-stimulating hormone (TSH), and human chorionic gonadotropin (hCG). These hormones share an identical alpha subunit, while their beta subunits are unique and confer hormone-specific biological activity. Both subunits are essential for the function of these hormones. The specific biological effects of FSH are primarily attributed to the beta subunit (FSHβ), which is also responsible for interaction with its corresponding receptor, the follicle-stimulating hormone receptor (FSHR) (29, 30).

Human FSHR is a member of the GPCRs family, characterized by a large extracellular domain (ECD), seven transmembrane domains, three short intracellular loops, three extracellular loops, and an intracellular tail (**Supplementary Figure 1**). FSH binding to FSHR occurs via the extensive ECD (29). The human *FSHR* gene, located on chromosome 2p21-p16, is a single-copy gene spanning 54 kb. Its structure comprises 10 exons and 9 introns, along with a promoter region. Notably, the extracellular domain is encoded by nine exons, whereas exon 10 encodes the C-terminus, transmembrane domain, and intracellular domain (29, 30).

Functionally, FSH plays a central role in mammalian reproduction, with its synthesis being regulated by gonadotropin-releasing hormone (GnRH) secreted by hypothalamic neurosecretory cells. FSH is crucial for development, growth, pubertal maturation, and overall reproductive processes. Specifically, it stimulates ovarian follicular growth during development in females and supports Sertoli cell function in males during adulthood. Interestingly, the FSHR is also expressed in various extragonadal tissues, including the placenta, uterus, prostate, bone tissue, and ovarian epithelium (31). The current understanding of diverse aspects of FSH biology and the FSH-FSHR interaction has been detailed in recent reviews (31, 32).

## 3 FSHR mutations and fertility

Various pathological conditions are known to be associated with FSHR structural defects caused by mutations, polymorphisms, and other alterations are known. Both activating and inactivating mutations in FSHR have been described in human, leading to alterations in reproductive function. The phenotype of these defects tends to be more severe in females, particularly affecting fertility (33–36).

To date, around 1800 SNPs in the *FSHR* gene have been reported, with two of the best characterized polymorphisms being rs6165 (c.919G>A; Ala307Thr) and rs6166 (c.2039G>A; Ser680Asn (**Supplementary Figure 1**) (27). These SNPs influence FSHR protein responsiveness to exogenous FSH, and have been shown to affect the success of *in vitro* fertilization treatment as well as the likelihood of developing severe ovarian hyperstimulation syndrome (OHSS). FSHR with haplotype Thr307-Asn680 possesses lower receptor sensitivity; as a result, higher FSH doses are needed for successful IVF treatments (37, 38). Importantly, recent Meta-analysis study revealed that the rs6166 Ser variant was significantly associated with an increased risk of poor ovarian response (POR), especially in Asian populations and the rs6165 Thr variant was significantly associated with an increased risk of POR, especially in Caucasian populations (39). Activating mutations in *FSHR* are associated with normal spermatogenesis in men but can lead to OHSS in women (36, 40). Some large studies have failed to confirm any association between the *FSHR* rs6166 genotype and serum FSH levels (35). Furthermore, this SNP exhibit ethnic-specific variations. Conforti et al. showed that rs6166 Ser/Ser carriers have higher basal FSH levels than Asn/Asn carriers and Ser/Ser carriers require higher doses of FSH for ovarian stimulation and produce fewer oocytes than Asn/Asn carriers. Thus, there is mixed evidence supporting an association between rs6166 and OHSS (36, 40). So far, the relevance of rs6166 for reproductive functions remains questionable, particularly in relation to ethnic phenotype. At first, the FSH-FSHR signalling was considered to exclusively regulate gonadal function, being a cornerstone of the hypothalamic-pituitary-gonadal axis. However, later several groundbreaking studies demonstrated the role of this hormone beyond reproduction, which will be discussed in the next paragraph (41, 42).

## 4 FSHR mutations and bone health

FSHR have been implicated in bone metabolism, with specific mutations associated with osteoporosis and low BMD (43, 44). In their work, Rendina and colleagues found that women carrying the FSHR rs6166 Asn/Asn variant had a higher postmenopausal osteoporosis risk than those with the Ser/Ser variant and that this SNP significantly influenced postmenopausal BMD (27). Postmenopausal women carrying the FSHR rs6166 Asn/Asn variant exhibited significantly increased levels of bone-specific alkaline phosphatase (BAP) and serum C-telopeptide of collagen type 1 (sCTX). They also experienced significantly decreased whole-body and femoral neck (FN) BMD, as well as lower calcaneus bone strength index. In agreement with other studies, these results were not influenced by circulating levels of FSH and estrogens (31, 35). An association between FSH and bone turnover markers (BTM) and BMD was confirmed for Chinese and Taiwanese women during the menopausal transition (44). These studies showed that changes in FSH levels were more strongly associated with bone turnover rates and BMD loss at the lumbar spine (LS) and total hip compared to estradiol (E2) or other hormones. This finding is further supported by data showing that in women during menopausal transition serum FSH level strongly correlates with the bone formation markers, such as BAP and osteocalcin (OC), and weakly correlates with the bone resorption markers, including sCTX and serum N-terminal telopeptide (sNTX), in women during the menopausal transition (45). However, in other similar studies, a weaker correlation between serum FSH and bone formation markers was found, which may be attributed to differences in ethnicity, sample size, and statistical models used. In a large multiethnic cohort study (Caucasian, African American, Japanese, Chinese) study, SWAN, involving 2, 336 women aged 42–52 years, Sowers et al. (2003) found that the relationships between FSH and FN, total hip, and LS BMD were negative, independent of ethnicity, physical activity, and body mass index (BMI) (46). Additionally, in pre- and perimenopausal women, Sowers et al. (2003) found that higher FSH levels were associated with higher sNTX levels and lower OC levels. Other sex hormones were not associated with the variations in bone remodeling markers (46). In another study, the relationship between FSH and BMD during ovulatory, anovulatory, and luteal phases of the ovulation cycle was studied in SWAN subjects of African American and Caucasian ethnicity. Their urinary FSH level was negatively and significantly associated with LS BMD at all three phases of ovulation cycle (47). All of the above support the view that higher FSH levels correlate with poorer bone health in premenopausal women. In general, accelerated bone loss in women during the menopausal transition ultimately leads to the manifestation of osteoporosis.

Human epidemiological studies have supported a concept of a negative relationship between FSH and bone health in perimenopausal women and elderly men, though this association was attenuated in postmenopausal women. The increased FSH levels observed in postmenopausal women play a major role in the pathogenesis of osteoporosis (41, 44, 46). As discussed earlier, the FSHR rs6166 Asn/Asn variant is associated with lower bone density, regardless of circulating E2 levels (27). Thus, the FSHR rs6166 Asn/Asn variant may be considered as a diagnostic tool for stratifying the risk of osteoporosis (24). Increased FSH levels are also characteristic of older men. Several studies have found a negative association between FSH with BMD in the LS, FN, and total hip, independent of testosterone levels, confirming the negative impact of FSH on bone density (48). In addition, large population-based study found no significant association between the *FSHR* rs6166 (Ser680Asn) polymorphism and BMD, bone loss, or fracture risk in elderly men and women. This negative result indicates that the effect of FSHR polymorphisms on bone health in the general elderly population may be too small to detect or may be masked by other, stronger determinants of BMD (e.g., age, BMI, vitamin D levels, other hormones (49). These findings are in a good agreement with data from the other study of postmenopausal women, which demonstrated that woman with the Ser680Ser (GG) genotype had a significantly higher prevalence of osteoporosis and lower BMD at the lumbar spine and femoral neck, as compared to those with the Asn680Asn (AA) genotype, suggesting that the less sensitive receptor (Asn680) appeared to be protective against bone loss (50). Hence, all above one may conclude that FSH’s action on bone is important and may be protective, but ligand concentration does matter. The association between FSHR genotype and bone mass is most apparent in the context of high FSH levels (42).

Recently, interest has increased in the role of FSH in bone metabolism in men (41, 48) as a metabolic role for the FSHR gene polymorphism rs6166 was shown. In healthy men, the Ser/Ser variant, associated with a less efficiently signalling FSHR, correlates with lower blood glucose levels compared to the Asn/Asn variant, which is a more efficiently signalling receptor. Men with the heterozygous Asn/Ser FSHR variant exhibited significantly lower insulin levels and HOMA-IR indices compared to Asn/Asn homozygotes, indicating a possible compensatory mechanism to prevent hyperglycemia (51). These findings were further supported by studies on Caucasian women, demonstrated that the FSHR rs6166 Ser/Ser variant may confer a protective effect against obesity, mirroring observations in men (50).

It has also been noted that the effects of FSH and E2 on BMD are not as strong as those of body weight and ethnicity (50). It is important to emphasize here, that FSH exerts an important effect on adipocytes (52). Large epidemiological data from the SWAN study have shown significant reductions in BMD and high resorption rates about 2–3 years prior to menopause, which were associated with increased body weight and visceral adiposity (53). In accordance, studies by Zhu and colleagues have shown that FSHR drives MSC commitment from the osteoblast toward an adipogenic lineage (54). This could be critical, as rising serum FSH levels may modulate the accumulation of visceral fat in post menopause and, thereby attenuate bone formation. A polyclonal antibody targeting a 13-amino-acid sequence within the receptor-binding domain of the FSHβ-subunit binds FSH specifically and blocks osteoclast formation *in vitro* (54). When injected into ovariectomized mice, this anti-FSH antibody not only inhibited bone resorption but also stimulated bone formation, thereby attenuating bone loss. Reduced fat mass has also been documented following treatment with a vaccine containing tandem repeats of the 13-amino-acid FSH receptor-binding FSHβ sequence (55). This highly conserved sequence, termed FSHβ13AA, serves as the FSH receptor-binding epitope in both humans and mice. These findings underscore FSH as a potential therapeutic target not only for preventing rapid bone loss but also for managing visceral obesity during the menopausal transition.

As discussed above, direct role of FSHR in bone biology highlights its translational relevance, as a considerable importance of FSHR in pathogenesis of osteoporosis has been demonstrated, particularly in postmenopausal women. The biological plausibility of FSH as a therapeutic target correlated well with the epidemiological studies that have shown a strong correlation between high serum FSH levels and low BMD alongside with increased fracture risk. Unfortunately, first-line anti-resorptive therapies involving long-term treatment with bisphosphonates is associated with rare but serious side effects (e.g., atypical femoral fractures, osteonecrosis of the jaw). A FSH-based therapy could intervene earlier in the pathological cascade. As discussed, elevated FSH-FSHR signalling activates bone resorptive cells, osteoclasts and their precursors. Hence, the primary therapeutic strategy consists of inhibiting FSHR, thereby reducing bone fractures. This strategy is supported by the evidence from the studies on OVX mice, which have shown that a specific anti-FSH antibody (MS-Hu6) completely prevented bone loss, increased bone mass, without affecting the reproductive axis. The MS-Hu6 showed the same level of “humanness” as human IgG1 *in silico* and was non-immunogenic in human peripheral blood mononuclear cell cultures. This approach is in active clinical development (56). Moreover, several small molecule inhibitors of FSHR have been identified through high-throughput screening (57) but their efficacy in bone models is still under evaluation. In summary, targeting FSHR is a highly promising strategy for improving bone impairment associated with accelerated bone resorption in patients with osteoporosis or slowing disease progression in postmenopausal women and potentially other groups.

## 5 Animal studies

**Figure 1:**
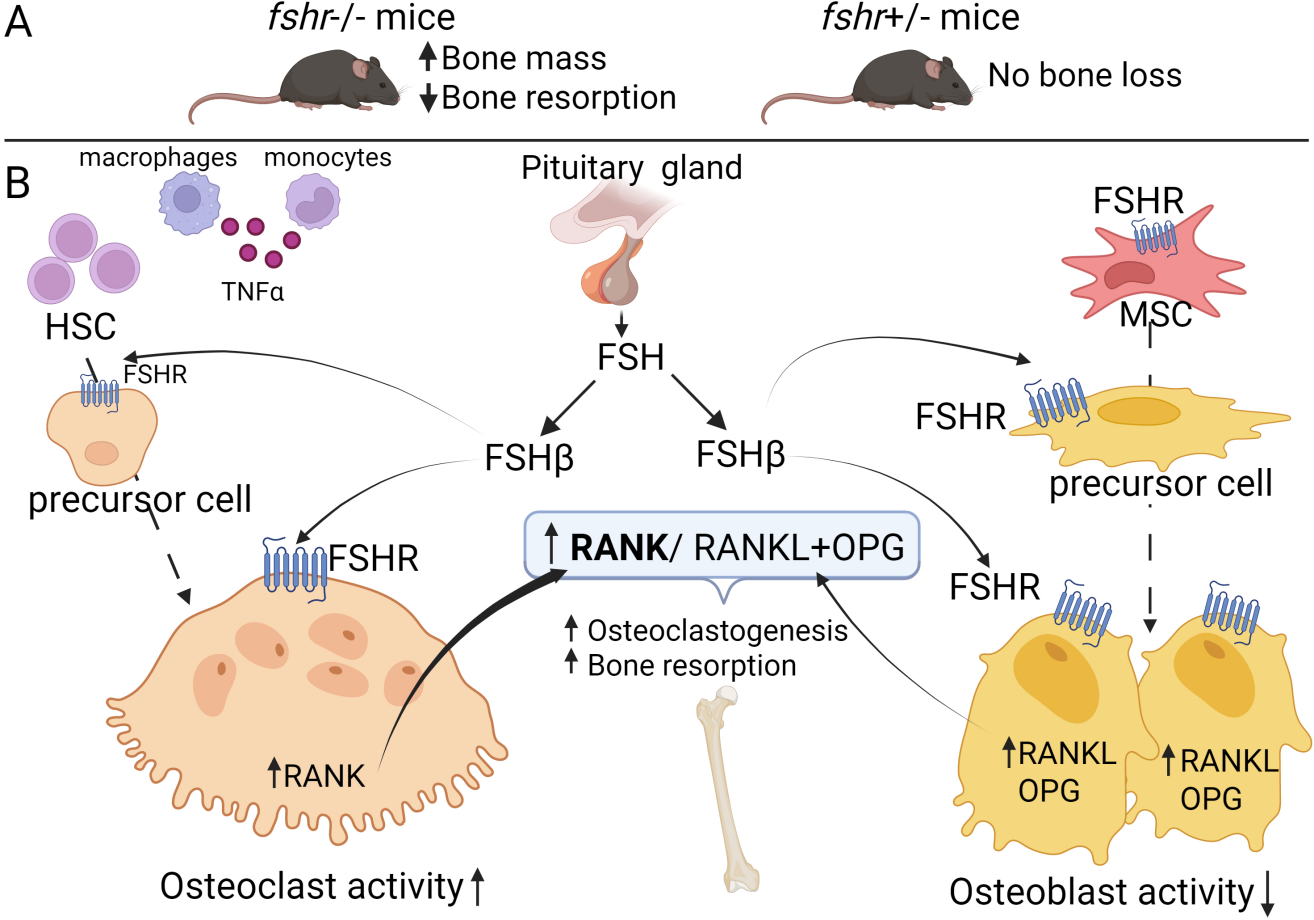
FSHR in bone biology. **(A)** Bone phenotype in *Fshr*-null and *Fshr*+/- mice: MSCs derived from Fshr-null mice exhibited elevated osteoblasts precursors colony suggesting pro-osteogenic effect of the *Fshr* knock-out. Fshr+/- exhibited no bone loss. **(B)** The mechanism of FSH-FSHR signalling in bone tissue. The pituitary gland derived FSH through its β-subunit, binds to FSHR expressed on both osteoblastic and osteoclastic precursors as well as mature bone cells. FSH exerts primarily catabolic effects on bone by promoting osteoclastogenesis through activation of the TNFα–NF-κB pathway. Additionally, FSH enhances osteoclast differentiation and maturation indirectly via upregulation of RANKL-RANK signalling (bold arrow). The role of FSHR in osteoblastogenesis remains controversial, with some studies suggesting potential anabolic effects while others report minimal direct involvement. Abbreviation: FSH, Follicle-stimulating hormone; FSHR, FSH receptor; HSC, hematopoietic stem cells; MSC, mesenchymal stem cells; OPG, osteoprotegerin; RANKL, RANK ligand; ↑­, upregulation; ¯↓, downregulation. Created with BioRender.

The direct effect of FSH on bone remodelling was studied *in vivo* through the genetic deletion of *FSH* or *FSHR* in mice. These studies demonstrated that while FSH contributed to hypogonadal bone loss, mice lacking either *FSHβ* or *FSHR* did not develop bone loss despite severe hypogonadism—an outcome that may be explained in part by elevated serum androgen levels. Notably, heterozygous *FSHβ+/−* mice with normal ovarian function, had 50% reduction in serum FSH levels and exhibited increased bone mass and reduced osteoclastic resorption, supporting the notion that the skeletal effect of FSH is estrogen-independent (**Figure 1A**) (42). Bone-resorptive cells, osteoclasts, and their precursors, possess Gi2α-coupled FSHRs that activate MEK/Erk, NF-κB, and Akt signalling pathways. Activation of these pathways enhances osteoclast formation, survival, and function, suggesting that age-related increases in FSH levels may directly induce bone loss by promoting bone resorption (58). In *in vivo* models, FSH injection led to increased bone loss, whereas administration of FSH inhibitors reduced bone resorption in ovariectomized rats (59, 60).

Thus, FSH exerts a direct effect on bone resorption, mediated by FSHR expressed on osteoclasts and their precursors. The effect of FSH on osteoblastogenesis was also supported by animal studies. While early studies suggested that, the FSH effect on osteoblasts is minimal this was most likely due to inefficient detection techniques in these studies. More resent investigations relied upon generation of Fshr-ZsGreen reporter mice under the control of Fshr endogenous promoter. This model allowed to demonstrate increased expression of FSHR in the osteoblast lineage cells (61). It was also shown that MSC isolated from mice treated with anti-FSH antibody exhibited greater osteoblast precursor colony counts, similar to mesenchymal cells isolated from homozygous *FSHR* −/− mice.

Overall, findings from transgenic rodent models have shown heterogeneous skeletal responses to FSH, potentially influenced by compensatory ovarian testosterone production in animals lacking FSH or FSHR. While FSH supplementation has been shown to impair bone integrity, blocking FSH activity appears to confer skeletal benefits.

## 6 FSH –FSHR signalling in bone cells

FSHR expression has been confirmed in human monocytic cells, osteoclasts, osteoblasts and MSCs, though at lower levels than in ovarian cells (42). However, the impact of the FSHR on mature osteoblasts remains poorly studied (31, 32, 62). Osteoclastogenesis from human mononuclear cell precursors can be stimulated in a FSH concentration-dependent manner. Since MSCs express FSHR, it was initially hypothesized that FSH might act via osteoblast precursors or stromal cells to promote osteoclastogenesis. However, cultures containing only CD11b^+^ cells (excluding stromal cells) demonstrated a full osteoclastogenic response to FSH, confirming that its pro-osteoclastogenic effects are mediated directly through FSHR on osteoclast-lineage cells.

The paper by Iqbal and colleagues (2006) revealed that in mice FSH induced the production of tumour necrosis factor alpha (TNFα) in monocytes and bone marrow macrophages, which further promoted the proliferation of osteoclast precursor cells (63). Thus, FSH stimulates both the formation and function of osteoclasts *in vitro* and *in vivo*. Key signalling pathways activated by FSH during osteoclastogenesis include toll-like receptor and interleukin-1 receptor-associated kinases, cell adhesion and survival pathways (e.g., TNFs/NF-κB/BCL-2), and cytoskeletal remodeling (62). Supporting these findings, Sun and colleagues demonstrated that FSH promoted the formation of tartrate-resistant acid phosphatase (TRAP) positive cells from various types of macrophages via FSHR activation (64). This process involves the phosphorylation of protein kinase B (Akt) and extracellular signal-regulated kinase (Erk), as well as the nuclear translocation of c-Fos; all these signalling events are essential for osteoclastogenesis (64). Other studies have shown that FSH-induced osteoclastogenesis may result from increased RANKL-RANK interaction (**Figure 1B**). Notably, Cannon et al. (2011) found that FSH at the physiological levels, similar to as seen in perimenopausal women, promoted the expression of receptor activator of nuclear factor κ-β (RANK) on human CD14+ monocytes (65). Furthermore, FSH increased the expression of osteoclast differentiation markers, as TRAP, MMP-9, and cathepsin K, in RAW 264.7 cells, in a dose-dependent manner (66).

Collectively, these findings demonstrate that FSH promotes both the proliferation and differentiation of osteoclast precursors into mature, bone-resorbing osteoclasts by directly engaging key signalling pathways and transcriptional mechanisms.

Although the role of FSHR in bone-forming cells, osteoblasts, remains less clear (**Figure 1B**), recent studies have shown that bone morphogenetic protein 9 (BMP9), a highly potent osteogenic factor, can upregulate FSHβ in mouse embryonic fibroblasts (MEFs) (67). Exogenous expression of FSHβ in MEFs significantly increased BMP9-induced ALP activity and the expression of key osteogenic transcription factors such as Runx2 and Osx, as well as late osteogenic markers like osteopontin (OPN) and OC. Additionally, FSHβ augmented BMP9-induced BMP/Smad signalling, as evidenced by increased phosphorylation of Smad1/5/8. These effects were suppressed by treatment with anti-FSHβ antibodies, suggesting that FSHβ may enhance BMP9 signalling through an FSH/FSHR/cAMP-dependent pathway in MEFs. Moreover, injection of FSHβ-transfected cells into the flanks of nude mice led to ectopic bone formation (67). However, more studies are needed to clarify these mechanisms and their translational potential for clinical application.

**Figure 2:**
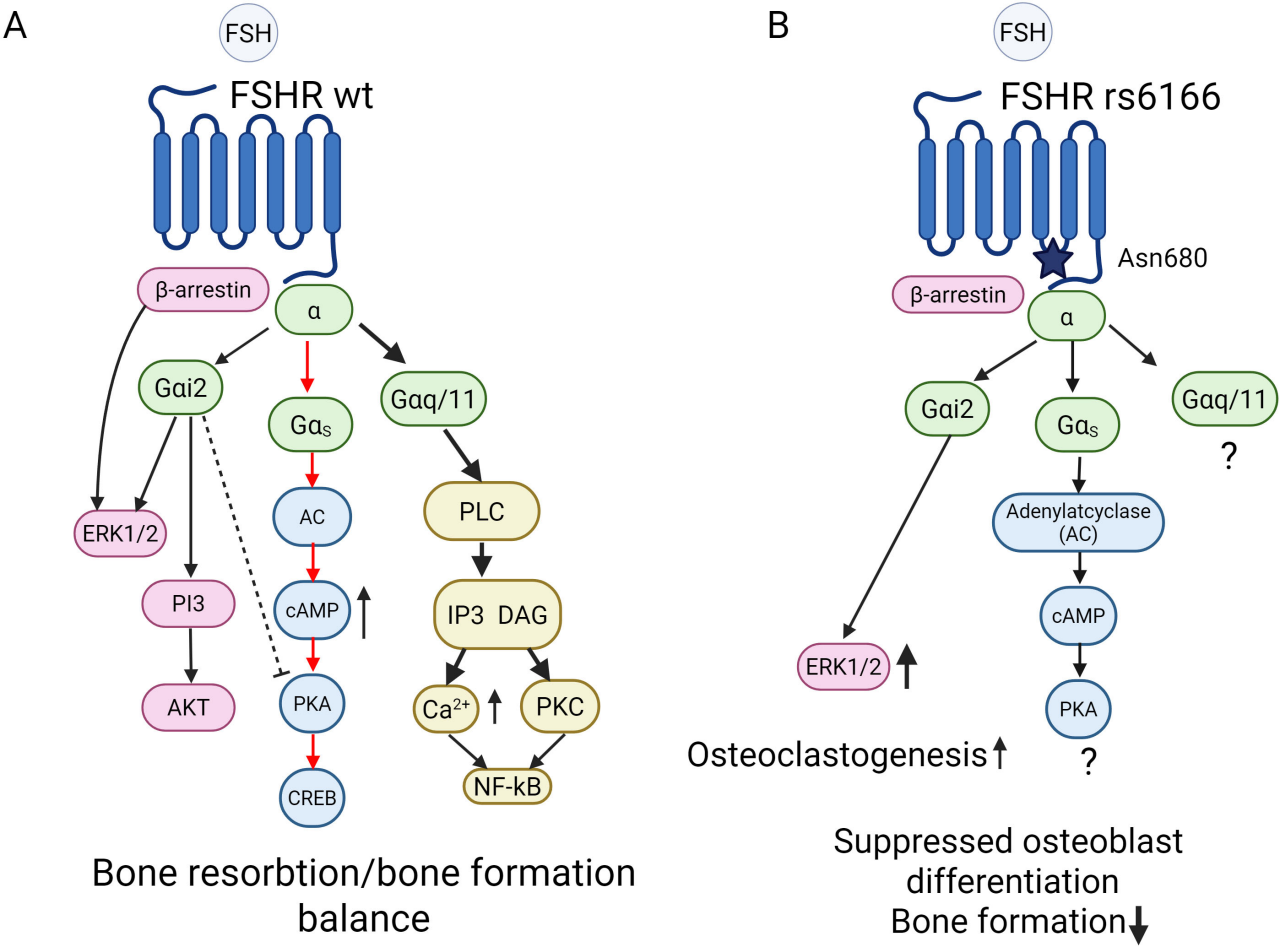
Simplified scheme illustrating the main pathways underlying the FSH- FSHR G-protein coupling leading to activation of the downstream signalling cascades in bone cells with wild-type FSHR (WT, **A**) and in rs6166 Asn680 **(B)**. **(A)** Balance in bone remodeling in WT cells is supported via the main signalling cascade from Gαs signalling (red arrows). In WT cells, the main Gαs-cAMP- protein-kinase A (PKA) pathway regulates target gene transcription, mainly via PKA and CREB. FSHR also interacts with β-arrestin, Gαi2 and Gαq/11-dependent pathways. Stimulation of Gαq/11 is important for intracellular Ca accumulation via PLC- IP3 pathway. Activation of PKC via Gαq/11 signalling supports NF-kB –mediated osteoclastogenesis. FSH- induced β-arrestin-dependent pathway important for the control of FSHR desensitization, recycling and activation of MAPK and ERK1/2 kinases. **(B)** In rs6166 Asn680 cells, signalling shift is suggested from Gαs signalling cascade towards pro-resorptive Gαi2 and Gαq/11-dependet pathway, leading to stimulation of osteoclasts activity, while suppressing osteoblast differentiation. Solid arrows represent facilitatory cascades, dotted line – possible inhibitory outcomes. Abbreviations: AC, adenylate cyclase: cAMP, cyclic AMP; IP3, inositol (1, 4, 5)-triphosphate; PLC, phospholipase C, DAG, diacylglycerol; CREB, cAMP response element-binding protein; NF-kB, Nuclear factor kappa **(B)** Created with BioRender.

It was mentioned before, the RANKL- RANK–OPG axis, is crucial for the osteogenic differentiation of MSCs and the regulation of bone metabolism. With employment of a human osteoblastic cell line Saos-2 it was shown, that RANKL is a target gene of PTH/cAMP/PKA signalling, meaning that cAMP/PKA cascade activates the canonical Wnt pathway (68). The Gαs - cAMP/PKA signalling is regarded as the primary signalling cascade for FSH-FSHR signalling via G-proteins and the main mechanism to promote osteoclastogenesis (**Figure 2**). Additionally, PKC activation via Gαq/11 may negatively regulate this effect by inducing expression of OPG (69). Osteoclasts, and their precursors, possess Gi2α-coupled FSHRs that activate MEK/Erk, NF-κB, and Akt signalling pathways (42). As discussed in (70) β-arrestins are also involved in FSHR signalling and internalization (**Figure 2**). Activation of the Gi2α –PI3-AKT pathway leads to the enhanced osteoclast formation, survival, and function. This implies that age-related elevated FSH levels may directly induce bone loss by promoting bone resorption via triggering G-protein alpha subunits signalling (58, 62) (**Figure 2**). Besides this, both the increase of the intracellular Ca^2+^ and the activation of PKC via Gαq/11 signalling support NF-kB –mediated osteoclastogenesis. As concerns the involvement of the FSHR-G-protein signalling in osteoblastogenesis, it is suggested that the RUNX2 –Osterix pathway is mainly inhibited by the Gi2α signalling and this leads to the inhibition of osteoblasts differentiation and, as a result, reduces bone formation (62). Of note, the ability of FSHR to engage multiple G-proteins is well-documented in gonadal cells. Generally, in GPCR pharmacology, it is well-established that a given polymorphism of a given GPCR may alter the receptor’s G-protein preference (71). While direct evidence of the ability of FSHR to bind multiple G-proteins in bone cells is limited it can be speculated that FSHR rs6166 (Asn680) polymorphism impacts on the bone cell physiology by attenuating the canonical Gαs/cAMP/PKA signalling pathway potentially biasing the receptor’s signalling towards Gαq/11 pathway, which is regarded to be more pro-resorptive (**Figure 2B**) However, FSHR signalling in bone cells is not limited to the canonical Gαs –cAMP pathway and may employ various G-protein coupling (**Figure 2**) highlighting the need for more research in this area.

In our work we have shown that the abovementioned homozygous SNP *FSHR* rs6166 (c.2039AA; Asn/Asn), associated with osteoporosis, might have direct impact on osteoblast differentiation (24). We have obtained three patient-specific cell lines from patient with osteoporosis, bearing this homozygous FSHR rs6166 Asn/Asn variant, and they all did show impaired osteoblasts maturation and mineralization capacity (24).

Overall, FSH has a direct effect on bone resorption, mediated by FSHR expressed on osteoclasts and their precursors. Studies in transgenic rodent models have yielded heterogeneous results regarding the skeletal effects of FSH, which may depend on ovarian testosterone production in FSH- or FSHR-deficient rodents. Supplementation with FSH in rats has been shown to negatively affect bone, whereas FSH inhibition appears beneficial. In humans, elevated FSH levels are generally associated with poorer bone health, particularly during the perimenopausal period. However, after menopause, the dominant effect of estrogen deficiency overshadows the impact of FSH, and the correlation between FSH and bone loss diminishes. Thus, FSH may partially explain the accelerated bone loss seen during perimenopause. Similar negative associations between FSH and bone health have also been observed in men.

## 7 TSH- TSHR biology

Thyroid-stimulating hormone (TSH) is a glycoprotein hormone secreted by the anterior pituitary that regulates the synthesis and secretion of thyroid hormones (THs). THs, in turn, regulate TSH secretion via a negative feedback mechanism (72). Thyroid hormone receptors (TRs) comprise TRα1, TRβ1 and TRβ2. Both TRα1 and TRβ1 are expressed in bone, with TRα1 being approximately ten times more abundant than TRβ1 (73).

The thyroid gland, composed of follicular cells and interfollicular C cells, produces THs: triiodothyronine (T3) and thyroxine (T4), as well as calcitonin. The production of THs is governed by the hypothalamic–pituitary–thyroid (HPT) axis, with approximately 80% of THs secreted as T4. A deficiency in THs can lead to symptoms such as fatigue, constipation, and weight gain, whereas excess levels are associated with cardiovascular disease and increased risk of osteoporosis (74).

As already noted, FSH and TSH are similar in their organization. TSH is also composed of two subunits: a common alpha subunit and a distinct beta subunit, responsible for biological specificity. Both hormones exert their effects through the cAMP second messenger system, although TSH can also activate the IP3/Ca²^+^ signalling cascade. Activation of both pathways enhances TH synthesis and promotes thyroid gland growth and differentiation (75).

TSH binds to and activates the TSH receptor (TSHR), which is expressed on the surface of thyroid follicular cells. Beyond the thyroid gland, TSHR is functionally expressed in osteoblasts and osteoclasts (76, 77), implicating TSH in direct action on bone remodeling. Activated TSHR undergoes phosphorylation and is internalized via clathrin-coated pits, where β-arrestin binds to the receptor and initiates β-arrestin–dependent signalling. TSHR preferentially utilizes β-Arrestin 2 for internalization in general, but in human osteoblasts, it interacts with β-arrestin 1 to promote differentiation and activate MAPK signalling (78).

In humans, the full-length TSHR protein comprises 744 amino acids with a molecular weight of approximately 87 kDa. It is encoded by the *TSHR* gene located on chromosome 14q31, which contains 10 exons (79). The first 9 exons encode the extracellular domain (ECD), while the 10th exon encodes the transmembrane domain (TMD) and a carboxyl-terminal region containing the intracytoplasmic domain (CD). Structurally, like other class A GPCRs, the TSHR exhibits a canonical topology (**Supplementary Figure 2**). A unique feature of the TSHR is its large N-terminal extracellular region, which comprises two critical subdomains: a leucine-rich repeat domain (LRRD) and a cysteine-rich hinge region that connects the LRRD to the transmembrane domain. Together, these extracellular regions govern hormone binding specificity and selectivity (80).

## 8 TH – TSH – TSHR signalling in bone cells

**Figure 3:**
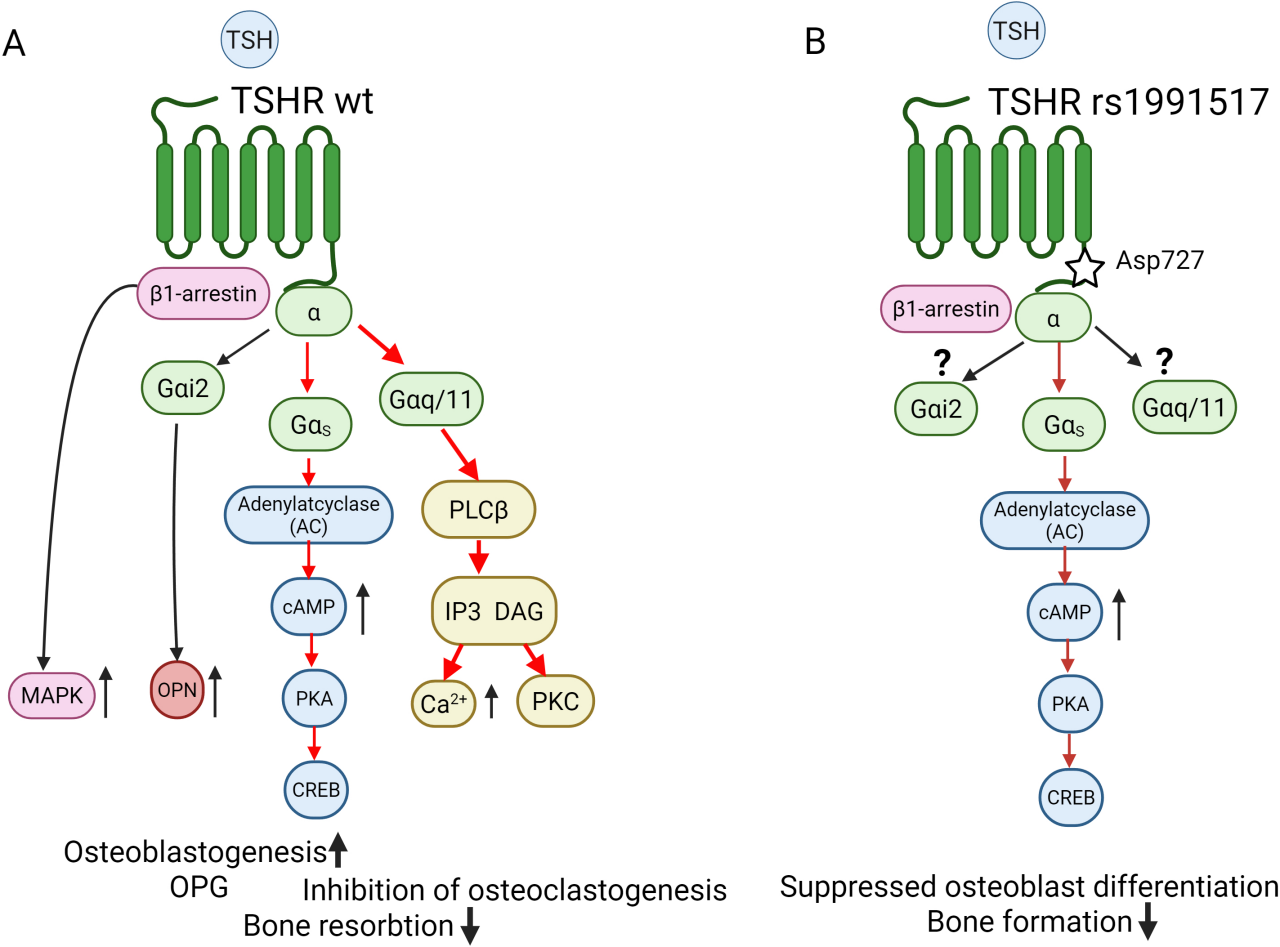
Simplified schematic presentation of the TSH-TSHR axis in bone remodeling via G proteins signalling cascades in **(A)** WT TSHR bone cells and **(B)** in TSHR rs1991517 Asp727 cells. **(A)** TSHR coupling to Gαs-cAMP-PKA signal transduction (red arrows) is considered to be a major pathway for osteoblasts differentiation and activity. TSHR is predominantly internalized by β-arrestin2, but β-arrestin1 signalling initiates both TSHR desensitization/internalization and MAPK activation, positively regulating osteoblast differentiation, supporting bone formation. Gαi signalling leads to phosphorylation of p38 MAPK and osteopontin (OPN) upregulation. High level of TSH activate Gαq/11-PKC-ERK1/2 signalling cascade supporting osteoblasts maturation. In addition, Gαq/11activates PLC, that cleaves PIP2 into DAG and Ca^2+^. Increased cytosolic Ca^2+^ promotes PKC translocation to the membrane and subsequent activation by DAG. In osteoclasts, Gαs-cAMP-PKA signalling inhibit osteoclastogenesis. Further explanation is given in a text. **(B)** Impaired Gαq/11 and Gαi2 signalling in osteoblasts leads to inhibition of RUNX2 and Osterix expression causing suppressed osteoblasts differentiation, while Gαs-cAMP-PKA signalling cascade supports osteoclasts activity and increases bone resorption. Solid arrows represent facilitatory cascades. AC, adenylate cyclase: cAMP, cyclic AMP; IP3, inositol (1, 4, 5)-triphosphate; PLC, phospholipase C, DAG, diacylglycerol; CREB, cAMP response element-binding protein; NF-kB, Nuclear factor kappa B. Created with BioRender.

TSHR is a classic GPCR and in bone cells, it primarily signals through two main G-protein subunits, such as Gαs and Gαq (**Figure 3**). Under stimulatory activation of the TSH-TSHR- Gαs –AC intracellular cAMP levels increases leading to PKA activation and phosphorylation of downstream CREB. This pathway plays an anabolic role and promotes osteoblastogenesis, while inhibiting apoptosis, thus supporting bone formation. Early works of Laugwitz and colleagues revealed that TSHR couples to several G proteins (81) to induce upregulation of genes associated with osteoblast activity. TSHR activates mitogen-activated protein kinase 1/3 (ERK1/2), p38 mitogen-activated protein kinase 1 (p38 MAPK), and AKT serine/threonine kinase 1 (AKT1) to support osteoblastogenesis (82, 83). It was shown that function of TSH as activator of TSHR signalling in bone cells is mediated through β-arrestin1. This pathway plays an important role in stimulating upregulation of osteoblast markers, such as ALP and OPN, supporting osteoblast maturation and differentiation (**Figure 3**). Knockdown of β-arrestin1, but not β-arrestin 2, inhibited upregulation of RANKL and OPN, thus supporting the evidence that β-arrestin1 signalling is important for osteoblastogenesis (78, 83). Additionally, Osteopontin (OPN) upregulation appears to be dependent on Gαi signalling, supported by TSH-mediated phosphorylation of p38 MAPK via Gαi (84). Another pathway that is important for osteoblast maturation is the regulation of ALP by TSH-TSHR. This regulation demonstrates a biphasic mode; in low concentrations TSH acts to downregulate ALP, which is mediated by the Gαs-cAMP pathway, whereas in higher doses TSH upregulates ALP, which is mediated by the Gαq/11-PKC-ERK1/2 signalling cascade (83).

However in osteoclasts, Gαs pathway plays an opposite role – it inhibits osteoclastogenesis via suppressing key osteoclastogenic factors like RANKL and NF-kB, while promoting the expression of the decoy receptor OPG. In addition, Gαs pathway can promote apoptosis in mature osteoclasts and thus inhibit bone resorption. Moreover, in osteoclast precursors TSHR signalling suppresses TNFα production via a Gαs –AC-cAMP- PKA pathway. Since TNFα is a potent stimulator of osteoclastogenesis, thus representing another anti-resorptive mechanism (85). Additionally, activation of Gαq/11, is generally leading to the release of intracellular Ca^2+^ and the activation of PKC (**Figure 3**). This pathway is involved in fine-tuning the osteoblasts differentiation. Overall, activation and signalling from TSH-TSHR via G-proteins performs fine-tuning of bone turnover and helps to preserve bone mass. This at least in part explains why conditions with high TSH (e.g., subclinical hypothyroidism) are often associated with higher BMD, and why low TSH (e.g., subclinical hyperthyroidism) is a strong risk factor for osteoporosis and fractures. The occurrence of SNP rs1991517 in TSHR gene results in the replacement of Aspartic acid (Asp) by Glutamic acid (Glu) at position 727 (Asp727Glu) in the intracellular tail of the receptor (**Supplementary Figure 2**). This region in the receptor is critical for G-protein coupling, receptor desensitization, and internalization (80). This genetic variant has been associated with lower bone turnover and higher BMD, primarily by causing a biased signalling effect that favors the inhibition of bone resorption over bone formation. Styrkarsdottir and colleagues (2008) demonstrated with a GWAS study that the rs1991517 variant is one of the first genetic factors significantly associated with BMD (86). Currently it is still not known how exactly the selectivity of a GPCR for a certain G-protein subtype is determined directly by the receptor (87). As discussed in Kleinau and colleagues (2017), TSHR selectivity on the intracellular site of the receptor can be altered by making an amino acid substitution that repulses a specific effector (biased inactivation) (80). Indeed, this mechanism is engaged by several inactivating mutations in the intracellular TSHR loops, for example, Gq activation is abolished by the Phe525Lys mutation (88). The fact that Gq-mediated signalling, but not Gs-mediated cAMP accumulation, can be impaired by single side chain substitutions in GPCRs suggests that Gq binding is more fine-tuned than Gs binding. However, the differences between Gs and Gq activation in bone cells bearing different SNPs in TSHR need further evaluation. In addition, more detailed pictures are needed of 3D structures of GPCR-G-protein complexes (80). Based on the available data, it can be suggested that in the case of TSHR rs1991517 Asp727Glu mutation, receptor signal towards Gαs, preserving the signalling cascade, rather than through Gαq/11, disturbing it. (**Figure 3B**).

Studies on mouse mutants with altered TSH or TH levels have revealed that the action of TSH on bone is likely minor compared to the effects of T3 (90). In bone cells, THs enter target cells via specific membrane transporters. The relative activities of type 2 and type 3 deiodinases (D2 and D3) are regulated to ensure optimal intracellular T3 availability. This leads to the displacement of co-repressors and recruitment of co-activators, thereby enabling the physiological transcriptional activity of TRα1—the primary receptor and mediator of T3 action in bone cells (72).

The importance of the TH axis in the regulation of skeletal growth and maintenance is well established through clinical studies. Data from genetically modified mouse models involving both disruption and overexpression of components of the TH axis components further support a concept of a key role for THs in bone metabolism. THs regulate the proliferation and differentiation of chondrocytes, osteoblasts, and osteoclasts. Their effects on target cells are mediated via ligand-inducible nuclear receptors, TRα and β, with TRα being particularly critical for bone cell function. Mechanistically, THs influence skeletal growth by modulating several key growth factor signalling pathways, including insulin-like growth factor-I (IGF-I), parathyroid hormone-related protein (PTHrP), fibroblast growth factor (FGF), Indian hedgehog (Ihh), and Wnt (89–91).

Triiodothyronine (T3) has been found to stimulate, inhibit, or have no effect on osteoblast proliferation; however, overall, it is believed that T3 stimulates osteoblast activity (92). *In vitro* studies using the mouse osteoblast-like cell line MC3T3-E1 demonstrated that T3 stimulation significantly increased the expression of osteoblast differentiation markers such as collagen I, OC, OPN, ALP, MMP9, and MMP13 (93). In addition, T3 modulates key pathways involved in osteoblast proliferation and differentiation and stimulates osteoblast responses to IGF1, PTH, and FGFs, both *in vitro* and *in vivo* (90, 91).

TH excess in mice causes high bone turnover, leading to bone loss. Supporting this, Ladermann and colleagues demonstrated that T3 treatment of primary murine osteoblasts enhanced their differentiation potential through activation of the BMP/Smad pathway, evidenced by phosphorylation of Smad1/5/8 (94). Conversely, another study using the osteoblastic UMR106 cells showed that THs inhibit osteoblast differentiation by suppressing the Wnt/β-catenin signalling pathway (95). T3 was also found to inhibit β-catenin pathway reporter gene activity in UMR106 cells co-transfected with TRa1 or TRb1. In the absence of TRs or T3, no such effect was observed, and a similar pattern was reported in MC3T3 osteoblastic cells. Thus, these finding indicate that T3 stimulates osteoblast activity through complex, both direct and indirect, mechanisms involving multiple factors and pathways.

The direct role of THs in stimulating bone resorption has been demonstrated in organ cultures of mouse calvaria (96) and fetal rat limb bones (97). Some studies have shown that T3 stimulates osteoclastic bone resorption in the presence of osteoblasts but not in their absence (98). These findings suggest that THs indirectly stimulate osteoclastogenesis by upregulating RANKL expression (99). *In vitro* studies using the mouse embryonic stem (ES) cell line WT 9.5 demonstrated that TSH supplementation during osteoblastic differentiation enhances Wnt5a secretion, promotes osteoblastogenesis, and increases mineralization capacity. Of note, TSH, through its action on frizzled (Frz) stimulates the production of OPG in ES cell-derived osteoblasts. This finding is significant, as OPG inhibits bone resorption by attenuating RANKL signalling (100). Interestingly, under these experimental conditions, TSH activated PKCδ rather than PKA, indicating that PKCδ is a downstream mediator of TSH action (101). The study proposed a feed-forward regulatory loop wherein TSH-stimulated Wnt5a production both enhances osteoblastogenesis and increases OPG secretion, which subsequently inhibits osteoclastic resorption to promote net bone formation (101). Osteoclast differentiation is regulated by the RANK/RANKL/OPG ligand-receptor system. OPG acts as a decoy receptor for RANKL, antagonising its interaction with RANK and thereby inhibiting osteoclast differentiation. Thus, the RANKL: OPG ratio plays a critical role in determining osteoclastogenesis. Consistent with Baliram et al. (101), another study using the ES cell model similarly demonstrated that TSH stimulation significantly increased OPG production, thereby verifying TSH’s direct action on osteoblast-lineage cells and linking TSH-induced OPG upregulation to impaired osteoclastogenesis (102).

Further insights into bone metabolism were gained from a study using MC3T3-E1 osteoblastic cells treated with T3, which demonstrated that T3 upregulated osteocalcin (*BGLAP* mRNA) expression. Interestingly, T3 only stimulated OPG expression in mature MC3T3-E1 cells, but not in their pre-osteoblastic counterparts, suggesting that the effect of T3 on OPG is differentiation stage-dependent. It appears, that in mature osteoblastic cells the 1, 25-dihydroxyvitamin D3 (1, 25D3), a hormone essential for skeletal maintenance and commonly prescribed for osteoporotic patients, inhibited T3-induced OPG expression but did not affect *OCN* mRNA level, whereas in pre-osteoblastic cells, 1, 25D3 completely inhibited both basal and T3-stimulated *OCN* mRNA expression. These results highlight the importance of balanced regulation of OPG and OCN transcript levels and suggest that T3 and 1, 25D3 collectively enable fine-tuned control of bone metabolism (103).

Taken together, these findings indicate that the effects of TSH on osteoblasts are still controversial. In Baliram’s ES cell model, TSH promoted osteoblast differentiation mainly by activating PKC and upregulating atypical Wnt pathway intermediates such as Frizzled 4 (Frz4) and Wnt5a (101). In contrast, other studies have shown that TSH can inhibit osteoblast differentiation and the expression of key osteogenic markers like type I collagen, bone sialoprotein, and OC, independent of Runx2 and osterix, by downregulating Wnt and VEGF signalling (104). Furthermore, Tsai et al. reported only low levels of TSHR expression, TSH binding, and cAMP activation in primary human osteoblast-like cells, speculating that TSH is unlikely to play a physiological role in the osteoblastic compartment of human bone (105).

Besides, TSH has been shown to inhibit osteoclast differentiation *in vitro*, as evidenced by reduced numbers of TRAP-positive cells and decreased expression of TRAP, MMP9, and cathepsin K in RAW264.7 cells (106), supporting the notion that TSH contributes to increased bone mass and strength at least in part by suppressing osteoclast formation.

Of note, the skeletal effects of THs extend beyond osteoblasts and osteoclasts, also regulating chondrocyte proliferation and differentiation through key growth factor pathways, including Indian hedgehog (Ihh), Wnt, IGF-1, and BMP/Smad (72). THs promote chondrocyte maturation and the progression of endochondral ossification, which is essential for longitudinal bone growth.

As detailed in subsequent section, murine genetic studies have yielded compelling mechanistic insights into TSH and TH regulation of skeletal development and bone remodeling.

## 9 Animal studies of TH – TSH – TSHR signalling

**Figure 4:**
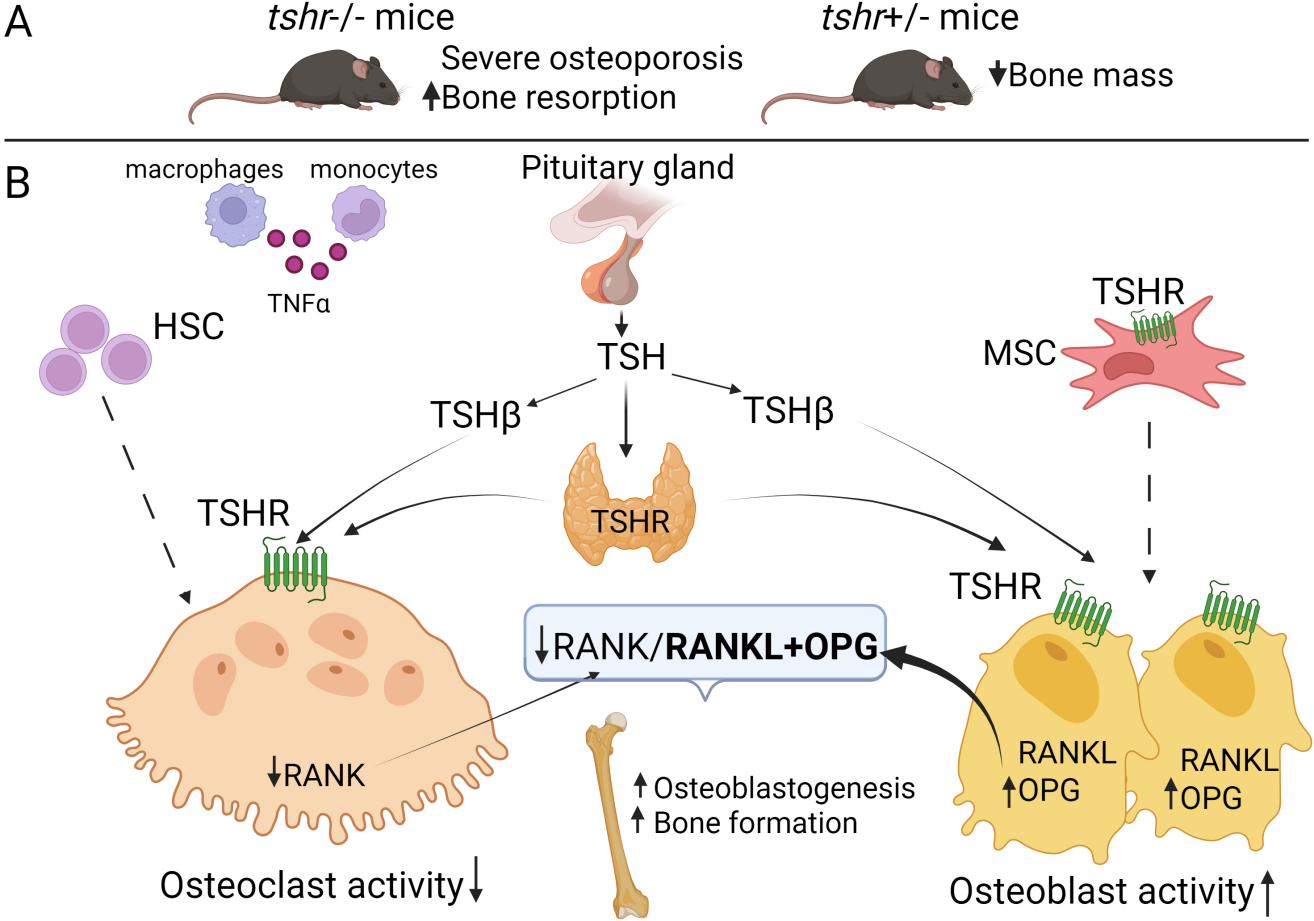
TSHR in bone biology. **(A)** Skeletal phenotype in tshr-null and tshr+/- mice. Tshr-null mice demonstrate a pronounced deterioration of bone tissue accompanied by enhanced osteoclastogenesis. Meanwhile, tshr+/- heterozygous mice also exhibit decreased bone density compared to wild-type **(B)** The mechanism of TSH-TSHR (anabolic) action on bone cells (tissue). The pituitary gland produces and releases TSH into the bloodstream. The TSH β-subunit, responsible for biological specificity, binds to TSHR expressed not only on thyroid follicular cells but also on osteoblasts and osteoclasts. In osteoblasts, TSH-TSHR signalling promotes their differentiation from precursors and enhances matrix mineralization capacity, while simultaneously stimulating OPG production – a natural decoy receptor for RANK ligand (RANKL) (bold arrow). This osteoblast-mediated action suppresses osteoclastogenesis. Besides, TSH exerts direct inhibitory effects on osteoclasts by reducing resorptive activity of mature cells, blocking precursor differentiation and reducing the production of TNFα attenuating NFκB signalling. Abbreviations: TSHR, thyroid-stimulating hormone receptor; TSH, thyroid-stimulating hormone; MSC, mesenchymal stem cells; HSC, hematopoietic stem cells; OPG, osteoprotegerin; RANKL, RANK ligand. Created with BioRender.

Mice lacking the *TSHR* gene (TSHR^-^/^-^) exhibit severe developmental and growth retardation, profound hypothyroidism, and severe osteoporosis (**Figure 4A**), characterized by undetectable serum T3 and T4 levels but elevated TSH. Without TH supplementation, these mice die within one week of weaning (107). However, even when treated with TH after weaning, TSHR^-^/^-^ mice failed to normalize their low BMD, indicating that bone loss is independent of circulating THs levels (104). Abe and colleagues (104) investigated the cellular basis of osteoporosis in these mice. They found a ~2-fold increase in osteoclast formation in cultures derived from both TSHR^-^/^-^ and TSHR^+^/^-^ mice compared to wild-type controls. Further *in vitro* analyses showed that TSH inhibited the expression of type I collagen, bone sialoprotein (BSP), and OC in primary calvarial osteoblasts and calvarial explants, while Runx2 and osterix (Osx) expression remained unaffected. In long bones of TSHR^-^/^-^ mice, the expression of LRP5 and Flk-1, factors associated with osteoblast differentiation was significantly upregulated, suggesting that TSH may inhibit osteoblast differentiation through Runx2- and Osx-independent mechanism. Additionally, TSHR^-^/^-^ mice exhibited increased levels of TNFα, a cytokine known to promote osteoclast precursor differentiation. In the absence of *TSHR*, osteoclast precursors showed enhanced RANKL-mediated differentiation and increased NF-κB activation, suggesting that osteoporosis in TSHR^-^/^-^ mice results from an imbalance between bone resorption and formation, with increased osteoclast activity.

Interestingly, heterozygous TSHR+/− mice despite being euthyroid with normal circulating levels of THs and TSH, still exhibit a significant decrease in bone mass. TH supplementation in these mice restores body weight but does not improve bone density (104), reinforcing the notion that TSH plays a direct role in regulating bone remodeling by acting on both osteoblasts and osteoclasts.

Thus, animal models provide compelling evidence for the distinct and essential roles of both TSH/TSHR signalling in bone remodeling and of THs in skeletal morphogenesis. The expression of RANKL and OPG in thyroid follicular cells (108) raises the possibility that the thyroid gland may regulate skeletal morphogenesis and remodeling in novel ways that warrant further investigation. Overall, TSH-TSHR axis appears an important regulator of bone homeostasis. Studies involving knockout mice have provided invaluable insights into this role.

## 10 Clinical manifestations

Williams and Bassett (76) provide a comprehensive overview of the skeletal consequences of thyroid disease in both adults and children in their article. Additionally, detailed insights into naturally occurring *TSHR* mutations, their associated clinical disorders, and the underlying molecular pathogenic mechanisms can be found in a comprehensive review (109).

In healthy individuals, circulating TSH and free T4 levels are maintained in inverse relationship through the hypothalamic-pituitary-thyroid axis, regulated by coordinated feedforward and negative feedback mechanisms (110). Hyperthyroidism, characterized by TSH concentrations below the normal reference range and elevated circulating T4 levels, is associated with reduced BMD and an increased risk of fractures, making it a well-established cause of secondary osteoporosis (111).

Loss- and gain-of-function mutations in the *TSHR* lead to TSH resistance with congenital hypothyroidism (OMIM 275200) or autosomal dominant hyperthyroidism (OMIM 609152), respectively. However, only a few studies have reported on the skeletal consequences of these genetic disorders (76, 112). In patients with congenital hypothyroidism who receive TH replacement, normal growth is restored and skeletal developmental abnormalities improve. Similarly, individuals with autosomal dominant hyperthyroidism show resolution of skeletal manifestations following thyroidectomy and normalization of thyroid status (76, 112).

TSHR mutations result in wide spectrum of clinical manifestations, ranging from mild to severe hypothyroidism and hyperthyroidism (113). More than 40 kinds of distinct loss-of-function mutations in the *TSHR* gene have been reported as causative defects in congenital hypothyroidism (114). Conversely, gain-of-function mutations in the *TSHR* gene have been identified in cases of familial or sporadic non-autoimmune hyperthyroidism (115).

Loss-of-function mutations in the TSHβ-subunit can also lead to TSH deficiency and congenital hypothyroidism. In primary hyperthyroidism, excessive production of THs by the thyroid suppresses TSH secretion through negative feedback. In primary hypothyroidism, the thyroid produces insufficient amounts of THs, leading to a loss of negative feedback inhibition and increase in TSH production. In secondary hyperthyroidism, the anterior pituitary secretes excessive amounts of TSH, which overstimulates thyroid follicular cells, resulting in elevated levels of THs. Conversely, in secondary hypothyroidism, reduced TSH production by the pituitary fails to adequately stimulate the thyroid, leading to decreased T3 and T4 levels. Subclinical hypothyroidism is defined biochemically by normal circulating concentrations of THs alongside an elevated TSH level. The impact of subclinical hypothyroidism on bone mineralization and fracture risk has not been extensively studied (64). In contrast, subclinical hyperthyroidism, characterized by suppressed TSH with normal THs levels, has been more clearly linked to adverse skeletal outcomes. Individuals with subclinical hyperthyroidism have shown increased bone loss and higher risk of fractures compared to euthyroid individuals.

A large meta-analysis involving over 70, 000 subjects showed that a TSH value below 0.01 mU/L is associated with a twofold increased risk of hip fractures and a 3.5-fold increased risk of vertebral fractures (111).

## 11 TSHR gene polymorphisms

A series of SNPs affecting the coding sequence of the *TSHR* gene have been identified. Some of them, such as rs61747482 (c.106G>C; Asp36His); rs2234919 (c.154C>A; Pro52Thr); rs1991517 (c.2181C>G; Asp727Glu), have been associated with susceptibility to autoimmune thyroid diseases (116, 117). The Asp36His and Pro52Thr variants have been found in both individuals with Graves’ disease and healthy controls. No significant difference in the frequencies of these alleles was observed between the two groups, suggesting that these TSHR polymorphisms occur at relatively high frequencies in the general population (117). Asp36His variant showed no significant association between healthy individuals and patients with osteoporosis (94). In contrast, the Asp727Glu variant was found to occur significantly more frequently in patients with osteoporosis compared to healthy controls. Logistic regression analysis demonstrated a significant correlation between the Asp727Glu genotype and both serum TSH levels and quantitative ultrasound measurements of the calcaneal bone. This was further supported by study in Brazilian patients with congenital hypothyroidism, which reported a high Glu727 allele frequency (10%) in affected individuals (116). A population-based study of approximately 1, 250 women from Scotland and followed for ~6 years confirmed a significant association between the Asp727Glu polymorphism and BMD (118). Homozygous Asp/Asp women had significantly lower LS and FN BMD compared to those without this genotype. Results remained significant after adjusting for confounding factors such as age, body mass index, menopausal status, and hormone replacement therapy use. Further analysis showed that the annual rate of LS bone loss in homozygous Asp/Asp women was approximately twice as high as in those without it (118).

Several clinical genetic studies involving mutations in genes related to the TH signalling pathway support a notion of an importance of THs and TSHR in regulating skeletal metabolism and bone remodeling. Human studies have shown a strong association between higher thyroid status, both within and outside the normal range and lower BMD. Several genetic studies have also demonstrated a relationship between TSH and BMD. Specifically, the homozygous TSHR Glu727 allele has been associated with lower TSH levels. In a study of 4, 934 elderly Caucasian men and women, carriers of the Glu727 variant had a 2.3% higher FN BMD. These findings point out to a positive association between serum TSH and BMD, and a potential link between the TSHR-Asp727Glu polymorphism and increased FN BMD (28, 119). However, as noted earlier, mouse genetic studies suggest that TSH and TH may exert independent effects on bone, with serum free T4 having a more pronounced influence on bone health than TSH.

Importantly, in another study, 706 common genetic variants have been mapped to the *TSHR* locus and its expression sites. However, none of these genetic variants were associated with BMD at the FN or LS (120). Hence, no evidence has been found that circulating TSH levels within the normal range are causally associated with abnormal BMD, nor is there any association between common genetic variations in the *TSHR* gene or its expression and BMD (120). Thus, the observed associations found in observational human studies between low circulating TSH and low BMD may be due to the reciprocal increases in free T4 levels, residual confounding, or reverse causality.

It is important to highlight that unlike the polymorphic variant of FSHR (rs 6166, Ser680Asn), the TSHR (rs1991517, Asp727Glu) variant has not been extensively studied *in vitro* in bone cell lineages (121). This emphasizes the need for further research using patient-specific cell lines that carry this SNP to understand its specific role in bone remodeling.

Discussion of the current clinical strategies of TSHR based therapy for osteoporosis should first of all call for the understanding of a direct role of TSHR in bone remodeling. TSHR expresses in both bone cells, osteoclasts and osteoblast and as discussed herein, TSHR acts as a negative regulator of osteoclast activity and a positive regulator of osteoblasts, leading to an osteoprotective effect. However, the effect of TSH in osteoblasts is still controversial and in some models TSH can inhibits osteoblast differentiation. That is often coupled with excessive resorption in high-turnover states. Therefore, this dual inhibition suggests that TSH can acts as a “brake” on the entire bone remodeling cycle. In states of hyperthyroidism this brake can be released, leading to accelerated bone loss. It is shown that postmenopausal women, men, and patients with a family history who receive TSH-suppression treatment have a high tendency to develop osteoporosis (122).

Diverse strategies have been developed of targeting TSHR, including monoclonal antibodies as well as peptides and small molecules. Some of them have been approved for clinical use, some of them are currently in different phases of clinical trials (123, 124). Development of small molecules or modified peptides, which can act as a biased agonists of TSHR, still remains in the preclinical discovery phase. However, this area is beyond the scope of the current review. In conclusion, the translational premise is that pharmacological modulation of the TSHR pathway in bone cells can re-establish control over bone remodeling, offering a novel therapeutic strategy for osteoporosis, particularly high-turnover osteoporosis. Key advances in this field are reviewed in the resent work by Zhang Y and colleagues (125).

## 12 Adrenergic receptors

Adrenergic receptors (ARs) are GPCRs that mediate physiological responses to catecholamines (e.g., epinephrine and norepinephrine) through G-protein-linked secondary messenger systems.

Advances in pharmacology and gene cloning have identified nine AR subtypes: three α1 (α1A, α1B, α1D), three α2 (α2A/D, α2B, α2C), and three β (β1, β2, β3). The genomic architecture of ARs varies: β1- and β2-ARs are encoded by intronless genes, while β3-AR and all α-ARs contain introns.

α1-Receptors are postsynaptic and coupled to G_q_ proteins. Their activation stimulates the inositol triphosphate (IP3) and diacylglycerol (DAG) pathways, leading to smooth muscle contraction and vasoconstriction of arterioles. α2-Receptors are primarily presynaptic and linked to Gi proteins, which inhibit adenylate cyclase, thereby reducing cAMP levels. Their activation suppresses norepinephrine release, induces vasodilation, and inhibits insulin secretion (126).

β1-Receptors are associated with G_s_ protein, increasing cAMP production. β1-AR are abundant in the cardiac tissues, where they play a key role in the cardiovascular function by increasing inotropy, chronotropy, conduction velocity. β2-Receptors mediate a broad range of effects, including smooth muscle relaxation in the respiratory and peripheral vascular systems. They also contribute to increased cardiac output through both chronotropic and inotropic actions. In the lungs, β2-AR activation promotes bronchodilation.

Recent studies using bone biopsies and primary human osteoblasts cell cultures have confirmed the expression of the three β-ARs in bone cells, with β2-adrenergic receptor (β2-ARs) being the most abundant, β1-ARs being less expressed, and β3-ARs being practically absent (127).

For decades, ARs have been recognized as a rich source for pharmacological exploration. Modulation of ARs by either activation or antagonism has yielded numerous therapeutic targets for pharmaceutical intervention. However, relatively few ligands can selectively distinguish AR subtypes. One of them is isoproterenol, a highly specific agonist for β-ARs. Propranolol is the most well-known antagonist for β receptors, and phentolamine is a potent antagonist for α receptors, through it binds weakly to β receptors.

For the purpose of this review, we will specifically examine the role of β2-AR and its polymorphisms in bone homeostasis and osteoporosis.

The β2-adrenergic receptor gene *ADRB2*, located on chromosome 5q31–32, spans a single exon of 2, 015 nucleotides encoding a 413-amino acid protein; notably, Kirstein and Insel identified at least 51 polymorphic sites within its 5.3-kb genomic region (126). As already mentioned for FSHR and TSHR, the classic β2-AR signalling cascade involves Gs protein-mediated activation of adenylyl cyclase, increasing intracellular cAMP. Although much of β2-AR biology has been attributed to its ability to stimulate cAMP production, numerous *in vitro* studies have demonstrated that β2-AR signalling and localization are intricately modulated by a variety of interacting proteins. These regulatory interactions fall into three main categories: (i) G proteins, such as Gs and Gi; (ii) protein kinases, including PKA, PKC, tyrosine kinases, and GRKs; and (iii) scaffolding proteins such as Arrestins, A-kinase anchoring proteins (AKAPs), and the Na+/H+-exchanger regulatory factor (NHERF) (128). The phosphorylation of β2-AR by GRKs, particularly GRK2 and GRK5, is known to enhance Arrestin binding, a key step in receptor desensitization and trafficking. Notably, receptor desensitization is also mediated by second messenger-dependent kinases such as PKA, and protein kinase C (PKC). PKA phosphorylates β2-AR at two distinct sites, diminishing its interaction with Gs and enhancing coupling to Gi, which leads to the release of βγ subunits and subsequent activation of MAPK signalling cascades. In osteoblastic cells, β-AR agonists have been shown to stimulate the production of RANKL, as well as other osteoclastogenic mediators such as interleukins IL-6, IL-11, and prostaglandin E (PGE), through pathways involving both PKA and p38 MAPK (129). Additionally, direct stimulation of osteoclasts by β_2_-AR activation has been reported, underscoring its functional relevance in bone resorption processes (130).

In the late 1990s, gene expression of α1 and 2 receptors (Adra1R, Adra2R) as well as β2-AR was detected in human periosteum-derived osteoblastic cells (SaM-1), human osteosarcoma-derived cells (SaOS-2, HOS, MG63), mouse primary osteoblasts, and human osteoclastic cells (131, 132). Among these receptors, β2-AR emerged as the primary functional adrenergic receptor in osteoblasts (133).

Adrenergic stimulation of the β2-AR influences cardiovascular function (134), highlighting its relevance as a key therapeutic target in cardiovascular disease. The clinical implications and pathological consequences of β2-AR dysfunction have been extensively reviewed elsewhere (135).

## 13 ADRB2 gene SNPs in health and bone

More than 250 polymorphisms in the *ADRB2* gene have been identified (136). These polymorphisms have been linked to altered receptor expression, down-regulation, and changes in cell signalling pathways *in vitro*. Among these, two nonsynonymous SNPs result in amino acid changes at positions 16 (rs1042713; c.46A>G; Arg16Gly, (**Supplementary Figure 3**); and 27 (rs1042714; c.79C>G; Gln27Glu), both of which have minor allele frequencies (MAF) between 40% and 50%. These SNPs are well characterized in asthma pharmacogenetics (137). *In vitro* studies have shown that the Gly16 isoform enhances agonist-stimulated down-regulation of β2-AR, while the Glu27 variant does not appear to affect receptor expression (138). The frequency of the rs1042713 Arg16 variant has been estimated at 39.3% in White Americans, 49.2% in Black Americans, and 51.0% in Chinese populations (139). Three meta-analyses have shown that the Gly16Arg variant is not associated with asthma, although the Gly16 allele has been linked to more severe forms of asthma (140, 141). Conflicting results have also emerged regarding the association of the Arg16Gly substitution with conditions such as type-2 diabetes mellitus, obesity, hypertension, and insulin resistance (142, 143). Studies conducted in Chinese populations suggest that the Arg16Gly variant may be associated with cholesterol metabolism (143). However, despite the long-standing investigation into the relationship between the Arg16Gly isoform and clinical outcomes in cardiovascular diseases, the data remain contradictory and warrant further research.

The estimated frequency of the Glu27 isoform of the *ADRB2* gene in White Americans, Black Americans and Chinese populations is significantly lower than for than Arg16 variant (139). The role of this polymorphism in heart failure revealed conflicting results (135). Association studies of the Gln27Glu variant with type-2 diabetes mellitus have also yielded mixed results, with some studies showing neutral, positive, and contradictory findings in various populations (144).

Moreover, no association between either rs1042714 or rs1042713 and obesity was found in a meta-analysis involving 23 populations. However, in race groups with low Glu27 allele frequency (such as Asians, Pacific Islanders, and American Indians), a significant association with obesity risk was identified for rs1042714 but not confirmed in East Asians for rs1042713 (145). Some reports have provided evidence that Glu27 variant is associated with a higher incidence of dyslipidemia and hypertriglyceridemia (146).

Nevertheless, as discussed above, the two common SNPs (rs1042713; Arg16Gly and rs1042714; Gln27Glu) of the *ADRB2* gene have been extensively studied. These variants may influence the development and outcomes of hypertension and other cardiovascular diseases. Emerging clinical evidence suggests that β2-AR genetic polymorphisms may serve as predictive biomarkers for therapeutic response in heart failure patients (147).

It is also important to note that early *in vitro* experiments revealed that neither agonist binding nor G-protein coupling, resulting in stimulation of adenylyl cyclase activity, was altered by the Arg16Gly or Gln27Glu polymorphisms. However, the Gly16 variant was associated with enhanced agonist-induced desensitization as compared to Arg16, while the Glu27 variant conferred resistance to desensitization (138). This resistance of the Glu27 receptor variant to agonist-promoted down-regulation was demonstrated in HEK293 cell lines over-expressing similar levels of either the Gln27 or Glu27 variant. Notably, the Glu27 variant appeared to enhance catecholamine-induced activation of ERK and p38 MAPK kinases. Given that these kinases are involved in myocyte hypertrophy, these findings may suggest a mechanistic link between this polymorphic variant and hypertension (148).

The Gly16Arg polymorphism also appears to influence outcomes in patients with heart disease. Specifically, individuals homozygous for Arg16 have been reported to have higher plasma norepinephrine and atrial natriuretic peptide levels, as well as increased left atrial diastolic dimension, compared to Gly16 homozygotes (149). Of note, in healthy individuals, Arg16 homozygosity has been associated with rapid agonist-mediated vascular desensitization, whereas Glu27 homozygosity correlated with enhanced agonist-mediated vasodilation (150). Moreover, in a hiPSCs derived cardiomyocytes Kondrashov and colleagues revealed that the reduced cAMP response can be attributed to a lower density of Arg16 receptor (151). Based on these and other findings, it has been suggested that the Arg16 variant of the β2-AR may represent a loss-of-function mutation, while the Glu27 variant may function as a gain-of-function mutation (151, 152).

Furthermore, a pivotal study by Kulminski et al. provided evidence that the Gln27Glu polymorphism, though not the Arg16Gly variant, of the *ADRB2* gene is associated with a broad spectrum of aging-related phenotypes, including various cancers, myocardial infarction (MI), intermittent claudication (IC), and both overall and healthy longevity. According to this study, the Gln27 variant increases the risk of cancer, MI and IC, whereas the Glu27 allele or the Gly16 Glu27 haplotype appears to confer a protective effect against these conditions (153).

Despite extensive research on β2-AR polymorphisms, their potential association with bone health and osteoporosis remains understudied, with only a limited number of publications addressing this relationship. For example, the Gln27Glu polymorphism has been associated with bone health. One study reported that SNPs in the Neuromedin U (NMU) gene, which regulates both energy metabolism and bone mass, as well as SNPs in the *ADRB2* gene, are linked to bone stiffness in children (154). Additionally, the frequency of inherited polymorphisms in the *ADRB2* gene and their association with rheumatoid arthritis (RA) have been shown in conjunction with the human leukocyte antigen (HLA)-DRB1 shared epitope (155). Furthermore, a study by a group of Spanish researchers demonstrated the direct impact of *ADRB2* gene polymorphisms on BMD (156). In their relatively small cohort of 61 women with normal BMD and 31 women with low BMD and osteoporosis, homozygous Arg16 genotype had significantly higher frequency in women with reduced BMD. These women showed an increased risk for reduced bone mass. However, no significant differences were found between the Arg16Gly and Gln27Glu polymorphisms and bone remodeling parameters, either in the general population or in those with normal or reduced BMD (156). These data are in concordance with our observation, that in a small cohort of osteoporotic patients the frequency of homozygous Arg16 genotype was about 17%, that doesn’t differ from expected population frequency.

The aforementioned studies are case-control meaning despite their robust sample size they may overlook critical details. In contrast, our own study, conducted on patient-specific cells lines revealed the association between impaired osteogenic differentiation *in vitro* and Arg16 variant of β2-AR. Specifically, we identified osteoporosis-associated SNPs in GPCR genes, which were linked to impaired osteogenic differentiation *in vitro* including *ADRB2* as well as *CNR2, MTNR1B, FSHR, TSHR, LGR4, CALCR and WLS* (24, 25). Further investigation of patient-specific cells homozygous for Arg16 variant of β2-AR revealed that altered osteoblasts differentiation is due to a disrupted proliferation-differentiation balance (25). Unlike case-control studies examining SNPs in osteoporosis, our approach of using patient-specific cell lines enables experimental validation of causality while minimizing confounding lifestyle and environmental factors. Thus, while case-control studies remain valuable for SNP discovery, research on patient-special lines underscores the necessity of cell lines models in elucidating the causal mechanisms underlying osteogenic dysfunction in osteoporotic patient derived cells. Through our work, we have highlighted the potential role of the Arg16 variant of β2-AR in disrupting osteogenic differentiation (25).

## 14 AR null animal phenotypes

**Figure 5:**
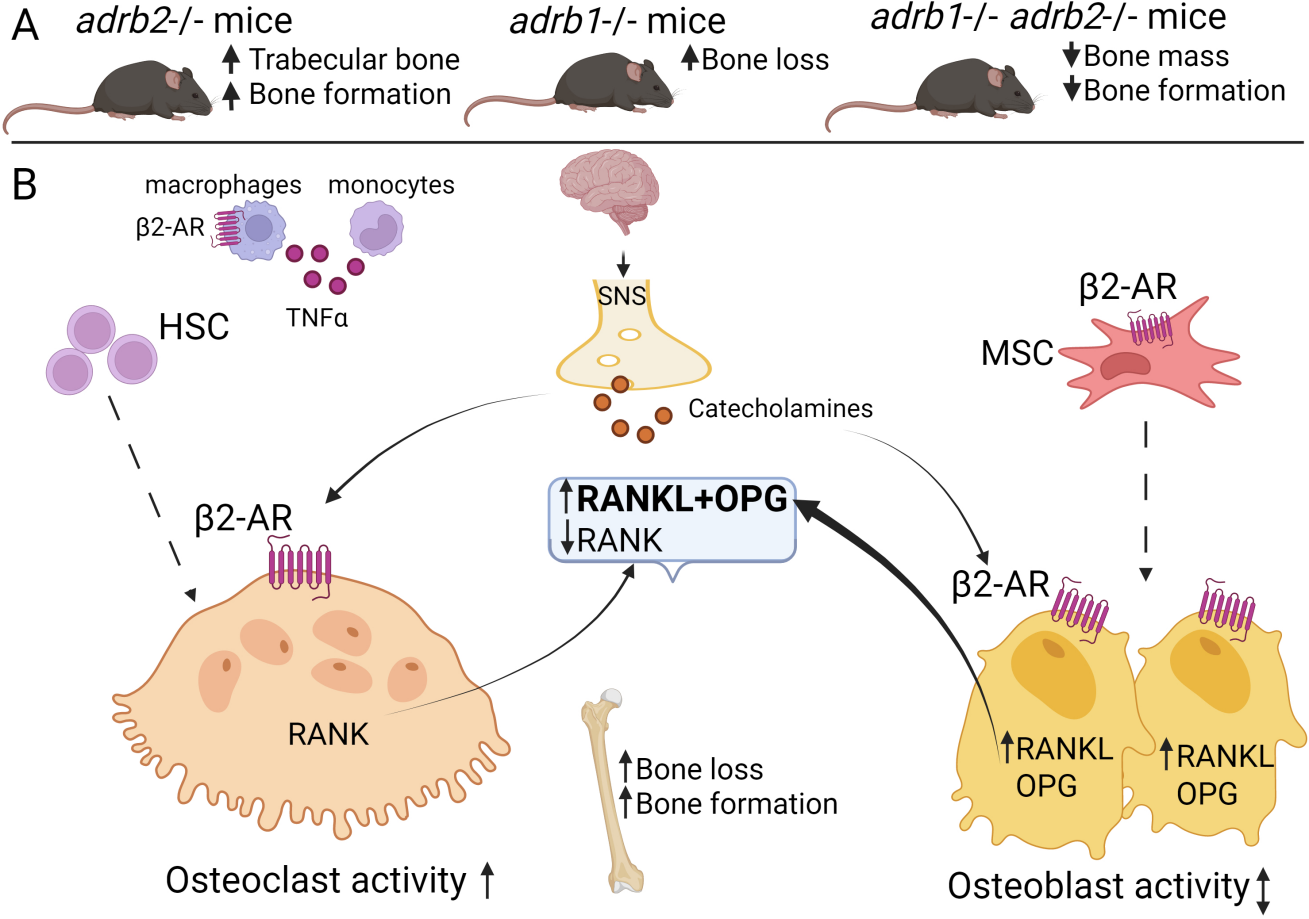
β2-AR in bone biology. **(A)** Phenotype of adrenergic receptors null mice. *Adrb2*-null mice show increased bone formation and decreased bone resorption, while *Adrb1*-deficient mice display the opposite phenotype with elevated bone loss. The double knockout of both receptors results in impaired osteogenesis. **(B)** The mechanism of β2-AR action in bone cells. β2-AR is activated by endogenous catecholamines (norepinephrine and epinephrine) and primarily promotes osteoclastogenesis through the RANKL-RANK pathway (bold arrow), leading to increased bone resorption. However, the effect of β2-AR activation on osteoblast function and bone formation remains controversial, with studies reporting conflicting results. Abbreviations: *adrb2*, beta-2-adrenergic receptor gene; *adrb1*, beta-1-adrenergic receptor gene; SNS, sympathetic nervous system; TNF α, tumor necrosis factor α MSC, mesenchymal stem cells; HSC, hematopoietic stem cells; OPG, osteoprotegerin; RANKL, RANK ligand, ↕, dual effect or conflicting results; ↑­, upregulation; ¯↓, downregulation. Created with BioRender.

β2-AR-deficient (Adrb2-/-) mice have exhibited an increased trabecular bone volume in the vertebrae and distal femur due to increased bone formation and decreased bone resorption (157). These mice have maintained normal body weight and hormonal status. Importantly, bone mass in these mice has remained unaffected following OVX, highlighting the essential role of intact SNS signalling in mediating estrogen-deficiency-induced bone loss. In contrast, β1-AR-deficient (Adrb1-/-) mice have been characterized by significant bone loss (**Figure 5A**) (157). Double β1-AR/β2-AR knockout mice (Adrb1/2^-^/^-^) have shown impaired bone formation, resulting in low bone mass (157). It should be noted that single gene deletions in the β-AR system may have led to compensatory upregulation or overstimulation of remaining receptor subtypes. For instance, Adrb1expression has been upregulated in adipose tissue of Adrb3^-^/^-^ mice, potentially limiting fat accumulation.

Collectively, findings from rodent knockout models and β-blocker studies support a regulatory role for the SNS, particularly β2-ARs, in bone metabolism (126, 158).

## 15 Role of β2-adrenergic receptors in bone homeostasis

It has been shown that the sympathetic nervous system (SNS) regulates bone metabolism and remodeling via β2-ARs. However, this function of β2-ARs remains less studied compared to their well-established role in cardiovascular diseases and other physiological systems. The use of β2-AR agonists and antagonists in *in vitro* models has contributed significantly to our understanding of β2-AR involvement in bone homeostasis.

**Figure 6:**
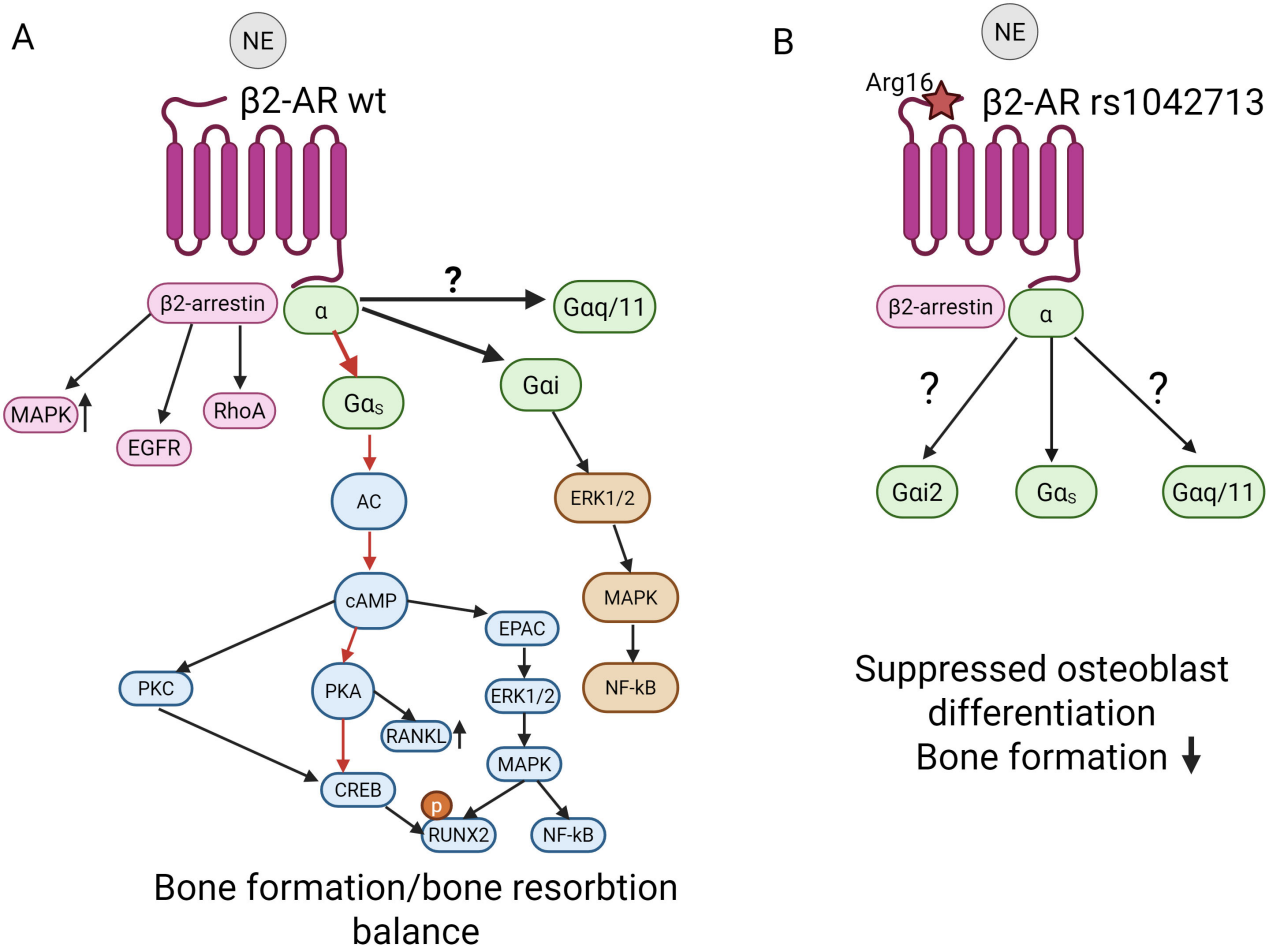
Simplified schematic representation of the main β2-AR-G proteins signalling pathways in bone cells for **(A)** WT β2-AR cells and **(B)** in β2-AR rs1991517 Arg16 cells. **(A)** β2-AR signalling activation in osteoclasts results in activation of the Gαs-AC-cAMP-PKA cascade and EPAC (exchange protein directly activated by cAMP), which triggers the activation of additional signal transduction pathways, further regulating cell survival, proliferation and differentiation. The activation of PKA results in the activation of transcription factors, including NF-кB and CREB family members. The phosphorylation of CREB by PKA or PKC leads to increased transcription of CREB target genes. In osteoblasts, the main pathways include Gαs/cAMP-PKA and Gαq/PLC-PKC, which activates RUNX2 and Osterix to stimulate bone formation. However, it is still questionable whether β2-AR in Gαq/11 signalling is responsible for the Ca^2+^ mobilization in osteoblasts. **(B)** The exact way of alteration in the G-proteins signalling in ADRB2 rs1042713 Arg16 is not known, but reduced Gαs-AC-cAMP activity was demonstrated, suggesting that pro-resorptive cascades can be more active in that case. Solid arrows represent facilitatory cascades. Abbreviations: AC, adenylate cyclase: cAMP, cyclic AMP; IP3, inositol (1, 4, 5)- triphosphate; PLC, phospholipase C, DAG, diacylglycerol; CREB, cAMP response element-binding protein; NF-kB, Nuclear factor kappa B; EGFR, the epidermal growth factor receptor; RhoA, Ras homolog family member **(A)** Created with BioRender.

The ability of GPCRs to signal through multiple G-proteins (Gαs, Gαi, Gαq/11) is well-documented (159). As already mentioned for FSHR and TSHR, the classic β2-AR signalling cascade involves Gs protein-mediated activation of AC, increasing intracellular cAMP (**Figure 6**). It was shown that β2-AR enhances RANKL expression in osteoblasts to amplify osteoclast activity. Hence, stimulation of β2-AR promotes the differentiation and maturation of osteoclast precursors *in vitro* and *in vivo* while inhibits proliferation and osteogenic differentiation of osteoblasts (160, 161). In osteoblasts, core pathways include Gαs/cAMP-PKA and Gαq/PLC-PK, which activates RUNX2 and Osterix to stimulate bone formation. Besides this, ERK 1/2 pathway has also been implicated in epinephrine-induced β2-AR signal transduction (162) (**Figure 6**). Inhibition of the PKA pathway, but not the ERK1/2 pathway, blocked RANKL expression in norepinephrine-stimulated MSCs and abolished their pro-osteoclastic effects (162). These results suggest that the β2AR-PKA axis is responsible for mediating the osteoclast-promoting potential of MSCs upon sympathetic stimulation. It is not currently known how the Arg16 rs1042713 mutation of the β2-AR alters the G-protein signalling. Like in the case of SNPs in FSHR and TSHR, the shift of the receptor affinity towards a preferential G-protein subunit may cause suppression of osteoblastogenesis, favoring osteoclastogenesis. The latter may be governed via Gαq/11-PKC and other pro-resorptive pathways, overriding the inhibitory cAMP pathway. This indeed can be the cause, as our data on the differentiation in the osteogenic medium of MSCs obtained from osteoporotic patients bearing rs1042713 Arg16 mutation demonstrated a complete absence of the Alizarin red staining in these cultures as well as an absence of the osteoblastic markers expression (25).

In primary osteoblast cultures, a dose-dependent increase in cAMP in response to the β2-AR agonist isoproterenol has been observed, which can be blocked by the non-selective β-blocker, propranolol. In osteoblast-like cells, β2-AR-mediated cAMP/PKA signalling leads to the expression of the immediate-early gene *c-fos*. Studies in rat bone cells and human osteosarcoma cells have demonstrated that c-fos forms heterodimers with jun proteins, regulating AP-1 responsive genes such as OC, ALP, and type I collagen (163). These findings suggest that β2-AR activation may promote osteoblast differentiation.

The effects of isoprenaline, a non-specific β-AR agonist, one bone metabolism have been also investigated in mice, with studies revealing divergent outcomes. One set of findings (164, 165), showed that chronic low-dose stimulation of the receptor induced bone loss primarily through enhanced bone resorption and decreased bone formation. However, another study (129) found that isoprenaline at certain doses affected only bone resorption, without comprising bone formation, highlighting a dose-dependent effect on bone remodeling. Additionally, research in ovariectomized rats demonstrated that low-dose propranolol, resulted in significant bone gain, whereas high-dose treatment did not yield the same benefit. Based on these results it has been proposed that β1-adrenergic signalling may promote bone formation and counteract the negative effects of β2-AR stimulation on bone formation (129).

However, clinical evidence remains inconsistent. In a small prospective study involving women taking propranolol daily, no significant changes in BTM were observed (166). Larger studies in postmenopausal women reported significant improvements in bone mass and reduced fracture risk among β-blocker (BB) users compared to non-users (167, 168). Further supporting a potential benefit, a 20-week trial in 165 postmenopausal women treated with BBs (propranolol, atenolol (β1-selective with partial β2 antagonism), and nebivolol (highly β1-selective)) demonstrated that atenolol and nebivolol significantly decreased the bone resorption marker CTx and the bone formation marker PINP, compared to placebo. A low dose of propranolol also reduced the bone resorption marker TRAP5b, an effect not seen with the more selective BBs. These findings suggest that in postmenopausal women, selective BBs may exert positive effects on bone health (126, 169). Nonetheless, longer-term studies are required to confirm these outcomes and to determine whether these effects arise solely from β-blocker action on osteoblasts or also involve modulation of bone vasculature.

Importantly, our study using patient-specific MSCs treated with propranolol revealed a pro-osteogenic effect of this beta-blocker. Specifically, MSCs exhibiting impaired osteogenic differentiation showed increased expression of *COL1A1* and enhanced alizarin staining, indicating improved matrix mineralization capacity following propranolol treatment (25). Thus, our research is one of the few to demonstrate the effects of beta-blockers on patient-specific osteoblasts, providing consistent evidence of their anabolic effects in humans.

Overall, several meta-analyses report a 28% reduction in hip fracture risk and a 14% reduction in overall fracture risk among BB users, indicating a possible protective effect on bone; however, findings across studies are not entirely consistent (170, 171).

The catabolic effects of β2-AR activation on bone metabolism are well established through *in vivo* experimental studies, which demonstrate that enhanced osteoclastogenesis leads to systemic bone loss mediated by RANKL upregulation. However, the impact of physiological catecholamines, such as adrenaline (epinephrine), on osteoblastogenesis remains underexplored. Interestingly, both pro- and anti-osteogenic effects of adrenaline have been reported (172, 173). The potential anabolic effect of adrenaline on bone formation may be linked to cyclic β2-AR stimulation at low doses (10–^9^ M), which promotes elevated cAMP levels (173). Given the reduced cAMP response associated with the Arg16 receptor variant, it would be valuable to investigate how physiological catecholamines influence osteogenic differentiation across different genotypes, considering the variations in cAMP response.

The exploration of the SNS, specifically the β2-AR, as a regulator of bone metabolism represents a significant progress in our understanding of bone biology. This “neuro-skeletal” pathway provides a novel, centrally-influenced target for osteoporosis therapy. The direct, functional link between the brain and bone stems for the translational relevance. As discussed above observational studies in humans taking BBs for cardiovascular conditions provided the initial clinical correlation. Multiple meta-analyses have reported that the use of non-selective BBs is associated with reduced risk of fracture and higher BMD (174, 175). Moreover, pharmacologically blocking β2-AR with specific beta-blockers offers a strategy to mimic the high bone mass phenotype observed in β2-AR-deficient mice, potentially leading to a novel anabolic therapy for osteoporosis. One of the major limitations of non-selective BBs is their cardiovascular (β1) and pulmonary (β2) side effects. Thus, the goal is to design a compound that selectively blocks β2-AR in bone while sparing receptors in other tissues. Despite the compelling preclinical and epidemiological data on BBs, significant challenges remain for clinical translation. A definitive, large-scale clinical trial is the crucial next step to move this promising concept from the bench to the bedside.

## 16 Conclusion and future perspective

Osteoporosis is a multifactorial disease and its clinical manifestation depends upon the complex interrelation between environmental factors, comorbidity and genetic factors. A thorough understanding of bone physiology, the mechanisms of bone remodeling, and the intricate signalling pathways involved is essential for the effective management of osteoporosis. Most patients with osteoporotic fractures present with multiple comorbidities, such as hypertension, diabetes, stroke, rheumatoid arthritis, and hyperthyroidism (176).

In this review, we discuss the data presented in the literature related to the role of the alterations in GPCRs gene functionality, namely *FSHR*, *TSHR* and *ADRB2* and their SNPs to elucidate the role of revealed SNP combination for bone homeostasis and osteoporosis. Our data on the small cohort of osteoporotic women have shown the prevalence of patients bearing homozygous FSHR rs6166 Asn/Asn, TSHR rs1991517 Asp/Asp and β2-AR rs1042713 Arg/Arg SNPs (24). Our extended unpublished data revealed increased allele frequencies for FSHR Asn680 - 0, 54 (in Russian North-West population frequency is 0.57); TSHR Asp727 -0, 93 (in Russian North-West population frequency is 0.86) and β2-AR Arg16 -0, 46 (in Russian North-West population frequency is 0.38), collaborated with clinical observational studies and pointing to the functional meaning of this three SNP combination with impairment bone homeostasis in osteoporotic patients.

**Figure 7:**
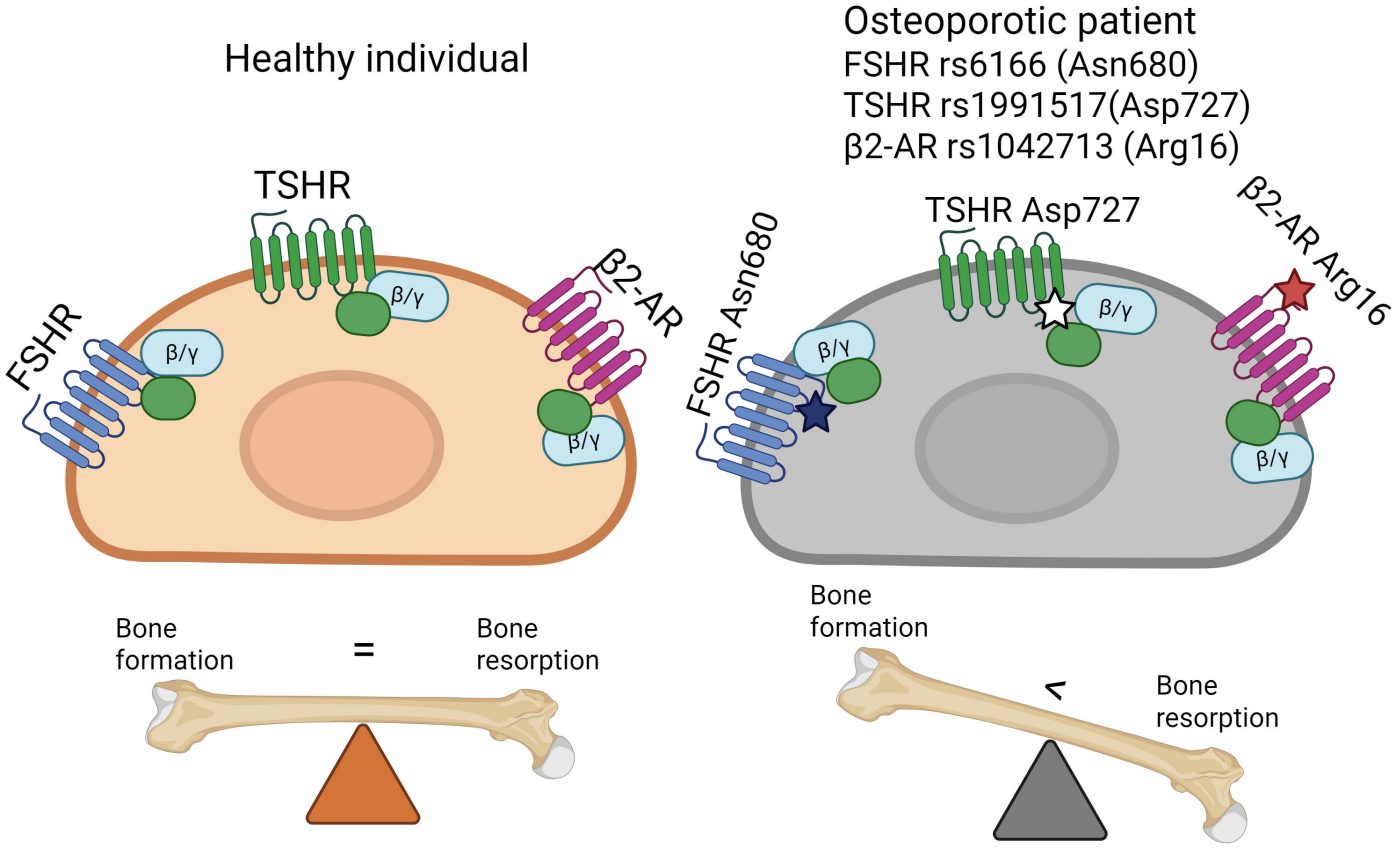
In healthy individual, bone remodeling remains balanced through normal receptor activity. Carriers of the SNPs which discussed in the present review, namely: FSHR rs6166 Asn680, TSHR rs1991517 Asp727 and β2-AR rs1042713 Arg16, may experience disrupted bone homeostasis with increased resorption, potentially leading to osteoporosis. These SNPs affect different receptor domains: FSHR rs6166 and TSHR rs1991517 influence the intracellular C-terminal domain, while β2-AR rs1042713 modifies the extracellular N-terminal domain. We propose that the FSHR rs6166 Asn680 variant is likely to be a gain-of-function variant, the β2-AR rs1042713 Arg16 variant appears to be loss-of-function variant, whereas the functional significance of TSHR rs1991517 Asp727 variant remains unclear. Created with BioRender.

Genetic risk factors of osteoporosis were addressed in numerous studies (177–179). Significant progress has been made to identify genetic variants and phenotypes associated with osteoporosis through GWAS (180). Efforts have been made to map associated variants to osteoporosis-causing genes (86, 181). However, individual SNP often only exhibit a small effect, but combinations of SNPs are assumed to be strongly influence the risk of disease. Still to reveal the causal one and to confirm via cell –line studies is challenging. We found that the carriers of the two SNP combination (TSHR rs1991517 Asp/Asp and FSHR rs6166 Asn/Asn) are highly prevalent in our osteoporotic cohort, and this is in good correlation with published data on FSHR rs6166 Asn/Asn variant, that is associated with lower bone density (27) and data on TSHR rs1991517 Asp/Asp variant, that is associated with lower LS and FN BMD in women (118). Little is known about the role of the β2-AR rs1042713 Arg16 on osteodifferentiation. Our data have demonstrated for the first time a detrimental effect of this SNP on osteoblastogenesis using patient-specific cell line (25). At the same time, as discussed here, the association of the β2-AR rs1042713, rs1042714, and rs1800888 with low BMD and osteoporosis is still debated (156). Based on the literature data discussed herein, high frequency of rs6166 FSHR Asn/Asn variant and rs1991517 TSHR Asp/Asp variant, and on our own data on rs1042713 β2-AR Arg/Arg (24, 25), we hypothesized that the combined contribution of all three SNPs leads to disruption of bone tissue homeostasis that ultimately leads to osteoporosis (**Figure 7**).

In addition to the key SNPs in the ADRB2, TSHR and FSHR genes, our NGS study of osteoporotic patients also revealed variants in other GPCR genes functionally linked to bone homeostasis: *CNR2* (rs2501431), *CALCR* (rs1801197), and *GIPR* (rs1800437). The rs2501431 (*CNR2*) polymorphism is associated with an increased risk of osteoporosis and lower BMD (182), suggesting a disruption of the receptor’s normal pro-osteogenic role (183). The rs1801197 (*CALCR*) polymorphism has also been linked to an elevated risk of osteoporosis in several studies (184), potentially impairing the receptor’s physiological function as a key inhibitor of osteoclast-mediated bone resorption (185). The rs1800437 (*GIPR*) variant is associated with an increased risk of non-vertebral fractures and lower BMD (186), consistent with the role of the GIP receptor in inhibiting bone resorption and promoting osteoblast survival (187). The concurrent presence of these SNPs suggests that the patient phenotype may result from a cumulative effect, where impairments across multiple nodes of the bone remodeling regulatory network exacerbate one another. Although the primary focus of this review paper remains on the *ADRB2-TSHR-FSHR* combination, these additional GPCR SNPs highlight the complex polygenic background of the pathology and provide a valuable foundation for generating hypotheses and planning future studies aimed at elucidating interactions within the entire receptor network.

What emerges from the studies discussed in this review is the realization that FSHR and TSHR have opposite effect on bone cells. TSH reduces the formation, function and survival of osteoclasts. This skeletal effect is dominant and convincing conclusion comes from the observation that mice with *tshr* haploinsufficiency are osteopenic (104), while subjects with gain-off –function SNP (TSHR D727E) displayed higher bone mass (28, 119). The idea that FSH stimulates osteoclast formation and function through FSH receptors on osteoclasts is consistent with the occurrence of high bone mass in *fshβ*-haploinsufficient mice (42). Mechanistically, both FSH and TSH interact reciprocally with MAP kinases, NF-κB and Akt kinases downstream of RANK-L, although the precise molecular cascades remain unclear. Importantly, the two hormones also share a reciprocal effect on the synthesis and secretion of TNFα: TSH inhibits TNFα production, whereas FSH stimulates it, actions that contribute to the anti- and pro-osteoclastic effects, respectively (63).

β-Adrenergic stimulation of bone resorption has been demonstrated in both *in vivo* and *in vitro* experiments (134). Osteoblastic cells induce osteoclastic differentiation and resorptive activity through the expression of osteoclast differentiation factor, namely RANKL/OPG/RANK. Epinephrine and isoprenaline have been found to enhance bone-resorbing activity of human osteoclast-like cells even in the absence of osteoblasts or stromal cells, indicating that β-Adrenergic agonists stimulate osteoclastogenesis both indirectly—via osteoblastic RANKL expression and directly, by acting on osteoclasts themselves (188). Our experimental data support the anabolic effects of the beta-blocker propranolol, demonstrating its ability to promote osteogenic differentiation *in vitro* (25). Taken together, data from pharmacologic, genetic and patient-specific cell line studies suggest that partial inhibition of adrenergic signalling, through low-dose β-blockers—may benefit skeletal integrity primarily by reducing bone resorption and improving bone formation. In contrast, non-selective or high-dose blockade, as achieved with high-dose propranolol or in *adrb1/2*^-^/^-^ double knockout mice, impairs bone formation and disrupts bone mineral balance. Moreover, postmenopausal woman display increased bone sensitivity to low doses of glucocorticoids which leads to activation of bone resorption and bone loss.

To summarize the above, we can conclude that SNPs rs6166 (FSHR), rs1991517 (TSHR), and rs1042713 (ADRB2) can interact synergistically to disrupt bone metabolism by converging and dysregulating shared intracellular signaling pathways, primarily the cAMP/PKA and MAPK/ERK cascades discussed here. The synergistic interaction between these three SNPs arises from their simultaneous involvement in disrupting key anabolic pathways and potentiating the catabolic pathway, each of which converges on shared intracellular signaling nodes. Indeed, FSHR and TSHR variants individually only modestly impair the important endocrine axis (gonadal and thyroid) that provides tonic support to the skeleton. Together, they create a state of reduced bone formation and increased bone resorption, leaving the skeleton more vulnerable to injury. Further to this, SNS signaling via β2-AR prove a potent catabolic signal, as it increases RANKL expression, stimulating osteoclast activity and suppressing osteoblast proliferation. The next step involves the integration of anabolic attenuation and catabolic potentiation of osteoclast activity. Thus, the skeleton, already weakened by impaired anabolic support from the FSHR and TSHR pathways, is exposed to a strong catabolic signal. The β2-AR Gly16 variant means that even normal sympathetic tone can eventually lead to a pathologically elevated skeletal response. Bone-forming osteoblasts, whose differentiation and activity are no longer optimal due to defective FSHR and TSHR signaling, are now directly suppressed by enhanced adrenergic input. This creates additional pro-resorptive condition, in which bone-building mechanisms are impaired, while bone-degrading mechanisms are accelerated. Next to this, the functional proof that rs1991517 itself is the causal variant and directly alters both human bone cell function is still lacking. Functional genomics studies using CRISPR-edited cell models and reporter assays are essential to confirm whether rs1991517 alters TSHR transcription, splicing, or expression. Further studies are needed to determine whether rs1991517 is a causal one polymorphism or it is simply only involved in linkage disequilibrium with the other true functional variant. Correlating genotype with TSHR expression levels in patients-derived bone cells would be a critical step. As discussed herein, the SNS via β2-AR, is a potent catabolic regulator of bone, inhibiting osteoblast activity and stimulating osteoclastogenesis through RANKL upregulation. The pathway is robustly supported by rodent models (126, 157) and human genetic associations (2). However, the data on patient-specific bone cell lines is very limited (25). Additionally, prospective pharmacogenetics studies are needed to determine whether the Arg16 variant predicts a superior skeletal response to beta-blocker drugs. Although, gene-environment interaction are important to clarify how this SNP interacts with lifestyle factors (e.g., chronic stress, exercise) to modulate fracture risk in different human populations.

The mechanism for maintaining skeletal homeostasis is based on the integrative nature of skeletal physiology, which includes not only the bone-brain axis and vice versa, but also the close interaction between all types of cells in the skeletal system, as well as the close interaction between the skeleton and other organs (189). Apparently, the identified SNP combination may cover a wide network of parameters regulating skeletal homeostasis and thus represent a new functionally significant combination for the diagnosis of osteoporosis. Certainly, more in-depth studies in osteoporotic patients of different ethnic соhorts are needed to confirm our hypothesis. In addition, expanding studies using patient-specific lines will help further move towards personalized medicine.

In order to represent the patient’s bone biological system, representative *in vitro* models require the patient’s own cells. However, the patient-specific experimental modeling approach faces several limitations. First, there is inherent genetic variability between patients: even when selecting donors/subjects based on shared genetic markers e.g. specific SNPs combinations, unaccounted genetic factors may still influence outcomes. Additionally, biological variables like donor gender, age, and comorbidities introduce another layer of complexity. Technical challenges present further obstacles — establishing pure monocellular cultures requires substantial effort of differentiation *in vitro*, while co-cultivation systems must carefully account for cellular compatibility and paracrine signalling. All such systems demand rigorous standardization and thorough validation. As promising solutions, researchers are actively developing organoid and 3D culture systems along with microfluidic platforms, which better replicate native tissue architecture and provide more physiologically relevant conditions (190).

Taking into account all of the above, it is necessary to conclude, that SNPs combination in ADRB2, TSHR and FSHR discussed herein, shows significant potential as an early diagnostic biomarker for predisposition to osteoporosis.

To robustly translate this finding into clinical practice, a clear validation pathway is required:

1. the immediate next step is to validate the predictive power of this SNPs combination in a large-scale, prospective cohort study. This would definitively establish its clinical utility by: determining the precise hazard ratio and population-attributable risk for developing osteoporosis based on an individual’s genotype; comparing its predictive accuracy against established clinical risk factors (e.g., FRAX score, BMD measurements) to evaluate whether it provides independent, additive diagnostic value.
2. a critical aspect of clinical translation is assessing generalizability. Our current findings are based on a cohort of postmenopausal women in Russian population and thus require further investigation in diverse ethnic cohorts. Future studies must specifically assess gender and menopausal status; it is essential to investigate whether this SNPs combination confers a similar risk in men and pre-menopausal women, given the distinct hormonal milieus across genders and reproductive stages. Since osteoporosis is a silent pandemic, it is likely that the search for such patients should be conducted among people with diabetes, obesity, cardio and hypertension health problems, since at the age of 45–55 years, patients notice these diseases earlier than disorders associated with bone disease.

In conclusion, we have identified a specific combination of SNPs in ADRB2, TSHR and FSHR that holds promise as a polygenic biomarker for the early diagnosis of osteoporosis. This could allow for the identification of at-risk individuals prior to significant clinical manifestation, enabling timely and personalized preventive strategies. The successful translation of this finding into clinical practice is contingent upon its rigorous validation in large, diverse cohorts and the subsequent development of a cost-effective diagnostic assay.
