# Supplementary material for: G-protein coupled receptors synergy in bone health: new avenues for osteoporosis detection and in vitro modeling
Source: Front Endocrinol (Lausanne). 2025 Nov 27;16:1684658. doi: 10.3389/fendo.2025.1684658 (PMC12695586; doi:10.3389/fendo.2025.1684658)
Supplement: Supplementary file 1 [file DataSheet1.pdf]

Supplementary Material

1 Supplementary Figures and Tables

1.1 Supplementary Figures

FSHR Asn680 FSHR Ser680

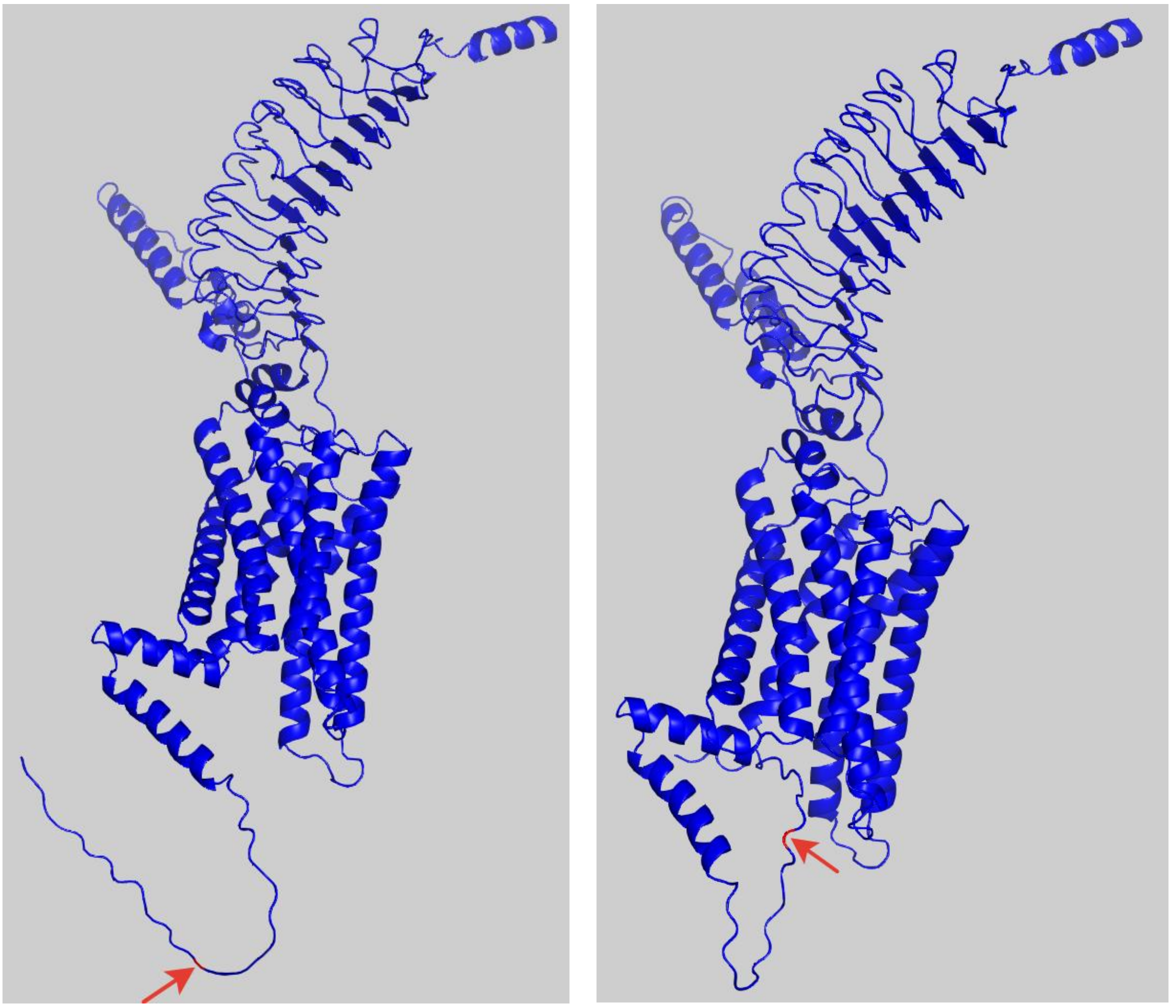

Supplementary Figure 1. Structures of FSHR proteins with either Asn or Ser in 680th position. Prediction made by Alphafold (https://alphafoldserver.com/), based on FSHR PDB structure 8I2H. The 680th position is colored in red and shown with red arrow. In both cases 680th amino acid is localized in intracellular unstructured region.

TSHR Asp727 TSHR Glu727

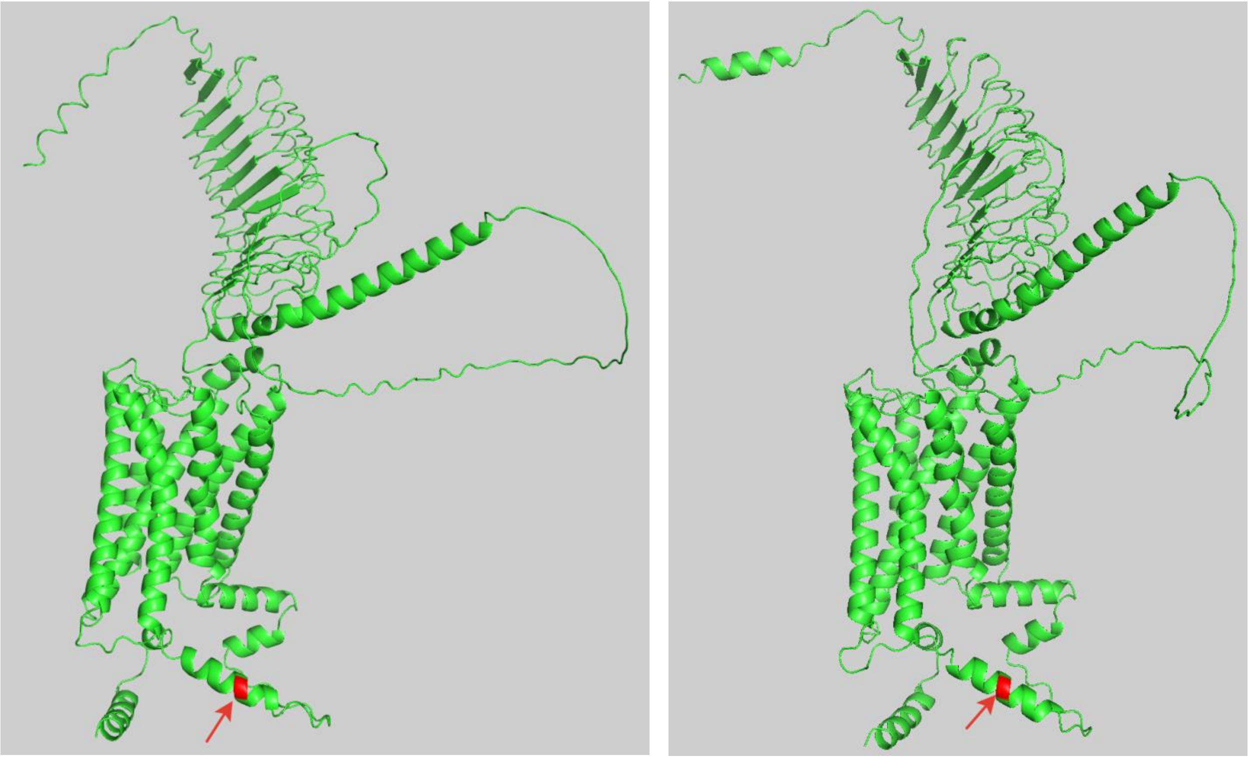

Supplementary Figure 2. Structures of TSHR proteins with either Asp or Glu in 727th position. Prediction made by Alphafold (https://alphafoldserver.com/), based on TSHR PDB structure 7XW7. The 727th position is colored in red and shown with red arrow. In both cases 727th amino acid is localized in intracellular α-spiral.

ADRB2 Arg16 ADRB2 Gly16

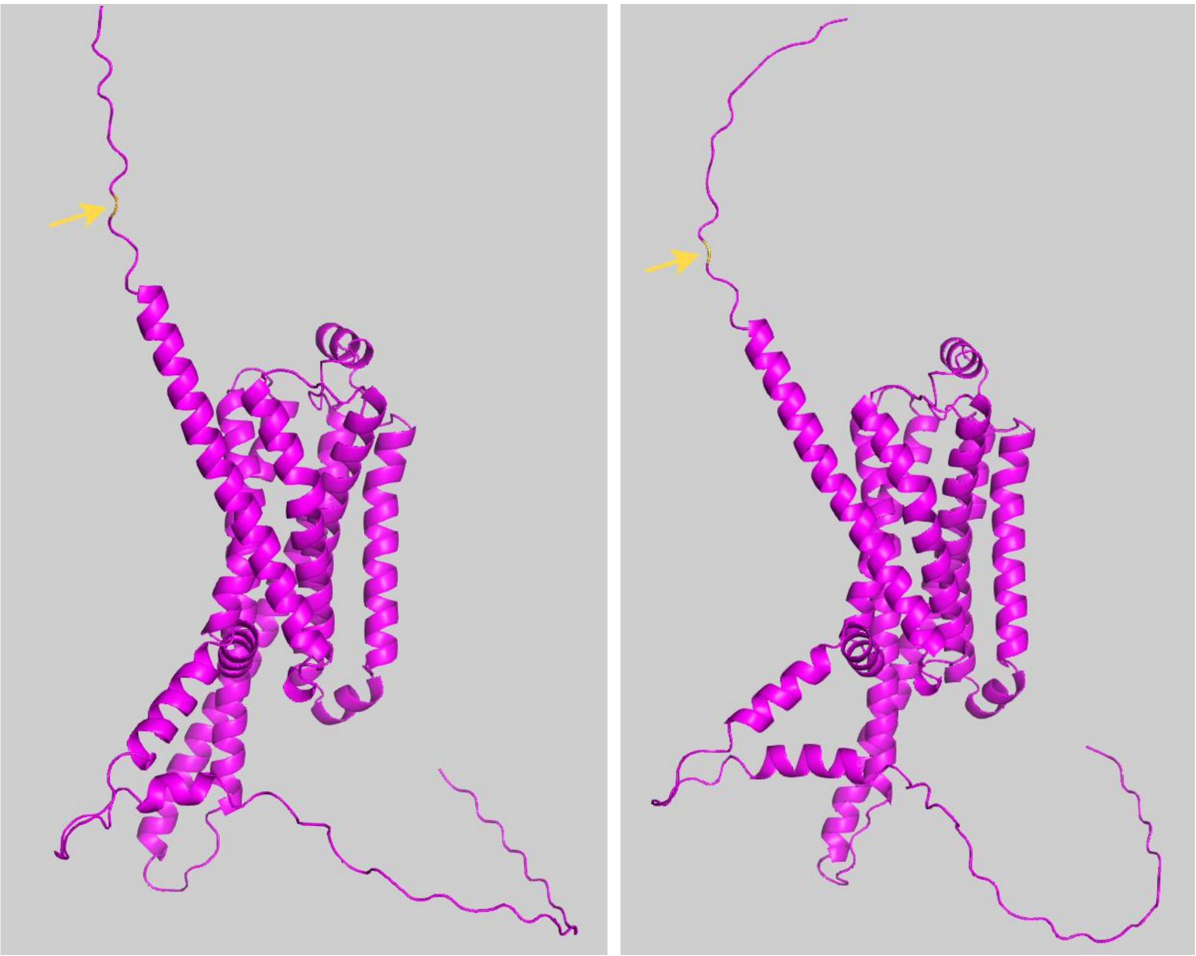

Supplementary Figure 3. Structures of ADRB2 proteins with either Arg or Gly in 16th position. Prediction made by Alphafold (https://alphafoldserver.com/), based on ADRB2 PDB structure 2RH1. The 16th position is colored in yellow and shown with yellow arrow. In both cases 16th amino acid is localized in extracellular unstructured region.

1.3. **Supplementary table 1.** Clinical findings for *FSHR* rs6166 (p.Asn680Ser), *TSHR* rs1991517 (p. Glu727Asp) and *ADRB2* rs1042713 (p.Glu16Arg) in connection to bone disease.

Clinical context .	Homozygous genotypes	
Postmenopausal BMD	*FSHR* rs6166<br>Ser680/Ser680<br>Less sensitive FSHR signaling<br>Higher BMD (Relative protection)	*FSHR* rs6166<br>Asn680/Asn680<br>More sensitive FSHR signaling<br>Lower BMD (Higher risk of bone loss)
Lumbar spine (LS) and femoral neck (FN) BMD in women	*TSHR* rs1991517<br>Glu727/Glu727<br>higher FN BMD	*TSHR* rs1991517<br>Asp727/Asp727<br>significantly lower BMD<br>faster rate of LS bone mass loss
BMD in women	*ADRB2* rs1042713<br>Glu16/Glu16<br>No effect	*ADRB2* rs1042713<br>Arg16/Arg16<br>Associated with reduced BMD

frontiers

Supplementary table 2. Comparative analysis between animal models and human data

SNP / Gene	Evidence from Animal Models	Evidence from Patient-Derived Cells	Comparative Analysis & Key Insights
**rs1042713 (*ADRB2*)**	**Strong, Mechanistic Evidence.**<br>**KO models:**<br>Adrb2-/- mice show a **high bone mass** phenotype, confirming the receptor's catabolic role (Pierroz et al., 2012).<br>**Agonist studies:**<br>Infusion of isoproterenol (β-agonist) causes bone loss in wild-type mice, mimicking chronic stress (Kondo and Togari, 2011).<br>**Limitation:** No precise "Gly16" knock-in model exists; models study complete receptor ablation, not the nuanced human polymorphism.	**Moderate, Correlative Evidence.**<br>• **Human airway smooth muscle cells:** Early studies showed enhanced downregulation properties of the Gly16 variant *in vitro* (Green et al., 1995).<br>**Mesenchymal stem cells:** Arg16 variant of β2-AR leads to altered osteoblasts differentiation is due to a disrupted proliferation-differentiation balance. Beta-blocker propranolol had pro-osteogenic effect (Krasnova et al., 2024)<br>• **Limitation:** Difficult to obtain primary human osteoblasts/osteoclasts in large, genotyped cohorts. The *in vivo* sympathetic tone is absent in culture.	**Synergy:** Animal models provided the causal proof that β2-AR signaling is catabolic. Patient cell studies confirm the variant is functional in humans. The key insight is that the Gly16 allele creates a hyper-responsive state *in vivo*, which animal models of global receptor loss perfectly align with.
**rs6166 (FSHR)**	**Controversial and Complex.**<br>**KO models:** Fshr-/- mice are infertile but show conflicting bone phenotypes, likely due to profound estrogen deficiency masking direct effects. FSHβ +/- mice demonstrated	**Emerging, Context-Dependent.**<br>**Granulosa Cells:** Robust data shows the Ser680 haplotype has **reduced cAMP response** to FSH (Nordhoff et al., 2011). This is the gold-standard functional evidence	**Contrast:** The clearest functional data for rs6166 comes from patient-derived granulosa cells, not bone cells or animal models. Animal models have been less informative due to the inextricable link between fertility and bone mass. The insight is that the primary functional impact is

	increased bone mass and reduced resorption (Sun et al., 2006)<br>**Cell-Specific KO:** Specific deletion of Fshr from osteoclast precursor cells significantly improved bone quality in mice (Chen et al., 2025).<br>• **Limitation:** The direct skeletal role of FSH is still debated; mouse models have not fully resolved the controversy.	for this SNP.<br>**Bone Marrow Cells:** Since FSH directly stimulates the formation and resorptive activity of osteoclasts (Sun et al., 2010), cells from Ser680 carriers may have a blunted resorptive response to FSH, but data is inconsistent.<br>• **Limitation:** Access to bone marrow cells from genotyped, pre- and post-menopausal women is a major logistical hurdle.	on gonadal response, with bone effects likely being secondary (endocrine) or more subtle.
**rs1991517 (TSHR)**	**Strong, for Pathway Validation.**<br>**KO models**: Tshr+/- mice are euthyroid but have **low bone mass** due to increased bone turnover, perfectly demonstrating the direct restraining effect of TSH on bone (Abe et al., 2003).<br>• **Limitation:** Models the *effect of reduced signaling* but does not validate the *rs1991517 SNP itself* as the causal variant.	**Indirect and Associative.**<br>• **Limitation:** The SNP's function is likely regulatory and tissue-specific, making it very difficult to capture in patient-derived cell assays.	**Divergence:** Animal models have been crucial in validating the *biological pathway* (TSHR signaling inhibits bone resorption). However, patient-derived cells have provided almost no direct functional evidence for rs1991517 itself. The association comes almost entirely from large human genetic studies (GWAS), and functional validation remains a major gap.

1. **Role of Animal Models:** They are unparalleled for establishing causal pathways and systemic physiology. The evidence for the role of *ADRB2* and *TSHR* in bone metabolism is strong primarily due to conclusive data from knockout mice. They provide a whole-organism context that cell cultures cannot.

2. **Role of Patient-Derived Cells:** They are essential for validating the specific molecular function of a human SNP in a relevant cellular context (e.g., *FSHR* in granulosa cells, *ADRB2* in MSCs and osteoblasts). They prove the variant has a functional consequence in humans.
3. **The Translational Gap:** For both rs6166 (*FSHR*) and rs1991517 *(TSHR*), there is a significant gap. We understand their strong association with osteoporosis from human genetics and their broad pathway from animal models, but we lack a clear, direct *in vitro* demonstration of how the SNP alters human bone cell function. For rs1042713 (*ADRB2*), the evidence is more complete, with both animal and human cell data converging on a coherent mechanism.

References

1. Abe E, Marians RC, Yu W, Wu X-B, Ando T, Li Y, Iqbal J, Eldeiry L, Rajendren G, Blair HC, et al. TSH is a negative regulator of skeletal remodeling. Cell (2003) 115:151–162. doi: 10.1016/S0092-8674(03)00771-2
2. Chen J, Liao Y, Sheng Y, Yao H, Li T, He Z, Ye WWY, Yin M, Tang H, Zhao Y, Zhang P, Wang Y, Fu X, Ji Y. FSH exacerbates bone loss by promoting osteoclast energy metabolism through the CREB-MDH2-NAD+ axis. Metabolism. (2025) 165:156147. doi: 10.1016/j.metabol.2025.156147.
3. Green SA, Turki J, Bejarano P, Hall IP, Liggett SB. Influence of beta 2-adrenergic receptor genotypes on signal transduction in human airway smooth muscle cells. Am J Respir Cell Mol Biol. (1995) 13:25-33. doi: 10.1165/ajrcmb.13.1.7598936.
4. Krasnova, O., Sopova, J., Kovaleva, A., Semenova, P., Zhuk, A., Smirnova, D., et al. (2024). Unraveling the mechanism of impaired osteogenic differentiation in osteoporosis: insights from ADRB2 gene polymorphism. Cells, 13:2110. doi: 10.3390/cells13242110
5. Kondo H, Togari A. Continuous treatment with a low-dose β-agonist reduces bone mass by increasing bone resorption without suppressing bone formation. Calcif Tissue Int. (2011) 88:23-32. doi: 10.1007/s00223-010-9421-9.
6. Nordhoff V, Sonntag B, von Tils D, Götte M, Schüring AN, Gromoll J, Redmann K, Casarini L, Simoni M. Effects of the FSH receptor gene polymorphism p.N680S on cAMP and steroid production in cultured primary human granulosa cells. Reprod Biomed Online. (2011) 23:196-203. doi: 10.1016/j.rbmo.2011.04.009.
7. Pierroz DD, Bonnet N, Bianchi EN, Bouxsein ML, Baldock PA, Rizzoli R, Ferrari SL. Deletion of β-adrenergic receptor 1, 2, or both leads to different bone phenotypes and response to mechanical stimulation. J Bone Miner Res. (2012) 27:1252-62. doi: 10.1002/jbmr.1594.
8. Sun L, Peng Y, Sharrow AC, Iqbal J, Zhang Z, Papachristou DJ, Zaidi S, Zhu L-L, Yaroslavskiy BB, Zhou H, et al. FSH Directly Regulates Bone Mass. Cell (2006) 125:247–260. doi: 10.1016/j.cell.2006.01.051
9. Sun L, Zhang Z, Zhu L-L, Peng Y, Liu X, Li J, Agrawal M, Robinson LJ, Iqbal J, Blair HC, et al. Further evidence for direct pro-resorptive actions of FSH. Biochem Biophys Res Commun (2010) 394:6–11. doi: 10.1016/j.bbrc.2010.02.113
